# Modeling the ribosomal small subunit dynamic in *Saccharomyces cerevisiae* based on TCP-seq data

**DOI:** 10.1093/nar/gkac021

**Published:** 2022-01-31

**Authors:** Tamar Neumann, Tamir Tuller

**Affiliations:** Department of Biomedical Engineering, Tel Aviv University, Tel Aviv 6997801, Israel; Department of Biomedical Engineering, Tel Aviv University, Tel Aviv 6997801, Israel; The Sagol School of Neuroscience, Tel-Aviv University, Tel Aviv 6997801, Israel

## Abstract

Translation Complex Profile Sequencing (TCP-seq), a protocol that was developed and implemented on *Saccharomyces cerevisiae*, provides the footprints of the small subunit (SSU) of the ribosome (with additional factors) across the entire transcriptome of the analyzed organism. In this study, based on the TCP-seq data, we developed for the first-time a predictive model of the SSU density and analyzed the effect of transcript features on the dynamics of the SSU scan in the 5′UTR. Among others, our model is based on novel tools for detecting complex statistical relations tailored to TCP-seq. We quantitatively estimated the effect of several important features, including the context of the upstream AUG, the upstream ORF length and the mRNA folding strength. Specifically, we suggest that around 50% of the variance related to the read counts (RC) distribution near a start codon can be attributed to the AUG context score. We provide the first large scale direct quantitative evidence that shows that indeed AUG context affects the small sub-unit movement. In addition, we suggest that strong folding may cause the detachment of the SSU from the mRNA. We also identified a number of novel sequence motifs that can affect the SSU scan; some of these motifs affect transcription factors and RNA binding proteins. The results presented in this study provide a better understanding of the biophysical aspects related to the SSU scan along the 5′UTR and of translation initiation in *S. cerevisiae*, a fundamental step toward a comprehensive modeling of initiation.

## INTRODUCTION

mRNA translation is a complex process consuming most of the energy in the cell (1–4) and affecting various fundamental biological processes and aspects (2,5–7). Thus, accurate modeling of this process has numerous applications (8–11). For many years, researchers aimed at modeling translation using small scale experiments and/or genomic data (3,7,12–20) that might result in biased conclusions as they do not measure translation directly.

**Figure 1.**
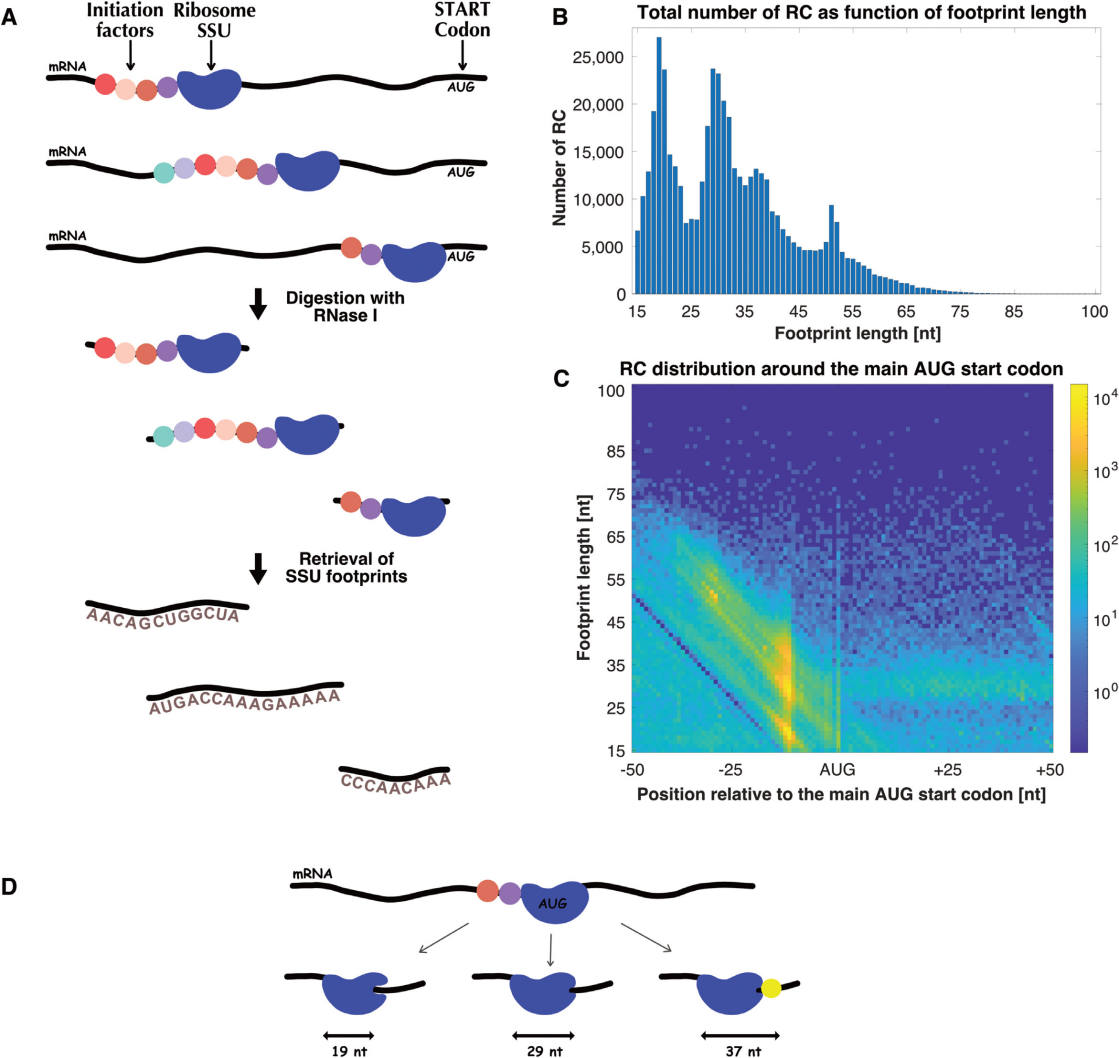
TCP-seq protocol provides the footprints of the SSU of the ribosome with additional initiation factors, resulting in RC distribution that has dependency both on SSU location in the 5′UTR and on footprint length. (**A**) TCP-seq protocol illustration. The scanning of the SSU is accompanied with additional initiation factors that promotes the movement toward the AUG start codon. The initiation complex size changes during the scan. The *S. cerevisiae* cells were crosslinked using formaldehyde that stall and attach the translation complex to the mRNA. Next, in order to generate protected mRNA fragments, the translation complexes were isolated and digested using RNase I. The SSU fractions were separated from the ribosome fractions using sedimentation velocity before the retrieval of footprints. Finally, the diverse sizes footprints were mapped to the *S. cerevisiae* genome. (**B**) RC histogram as function of footprint length, ranges between 15 and 100 nucleotides. It can be seen that most of the RC are up to ∼75 nucleotides. (**C**) The footprint 5′ends relative to the main AUG start codon are plotted versus footprint length, presenting the RC distribution around the main AUG start codon. The color scale corresponds to the number of RC, displayed in logarithmic scale. The dependency on both the location in the 5′UTR and both on footprint length creates a gray scale image that later was discretized in order to perform computational analyses. (**D**) Different SSU configurations near start codon, an illustration. SSU footprints coalesced into three major sizes at start codons—19, 29 and 37 nucleotides, mainly due to dynamic rearrangements at the entry to the mRNA channel—from an open to a closed state following start codon recognition.

Many translation models (see, for example (21–25)) have been enabled by the establishment of the Ribo-seq approach (2). However, since Ribo-seq is based on the generation of footprints related to the complete ribosomes during the elongation step, these models focus on elongation. Recently another approach, TCP-seq (26), was suggested, aimed at monitoring the movement of the small subunit (SSU) of the ribosome accompanied by initiation factors during the initiation step. Thus, this novel type of data can now be used for the first time for understating and modeling initiation, a rate limiting phase of the translation process (27). Nevertheless, the TCP-seq data are very challenging due to varying read lengths caused by various conformations of the SSU and the relevant factors in different positions along the mRNA (Figure 1).

Translation initiation in eukaryotes involves the binding of the pre-initiation complex (consisting of the small (40S) ribosomal subunit loaded with initiation tRNA) to the mRNA near the 5′ end. The pre-initiation complex, accompanied by additional initiation factors, scans the 5′UTR toward its 3′ end until recognition of the START codon (usually an AUG codon) (28–30). It was suggested that a particular context around the main AUG is required for the pre-initiation complex to recognize it and initiate translation (28). However, the aforementioned scanning mechanism and the dynamics of the SSU scan are not completely understood. For example, although the scanning mechanism predicts that initiation will occur at the nearest 5′end AUG codon, there are reported cases of leaky scanning (29), where the pre-initiation complex skips AUG codons with sub-optimal context and initiate translation farther downstream. In addition, a previous study showed that in many cases an AUG in the 5′UTR with relatively optimal context score doesn’t initiate translation (31).

Additional features are considered to affect the SSU scan, such as the mRNA secondary structure, as it was previously shown that the presence of strong secondary structures in the 5′UTR can significantly reduce protein levels (32–37). This inhibitory effect presumably stems from the ability of the pre-initiation complex to disrupt base pairing only to a certain limit, and to a lower extent than that of the 80s ribosome (38,39). Upstream open reading frames (uORF) also have a negative impact on translation efficiency as they engage the SSU before it reaches the main AUG start codon (40,41). It was reported that four major uORF properties are associated with greater inhibition: (i) strong upstream AUG (uAUG) context, (ii) evolutionary conservation, (iii) increased distance from the cap and (iv) multiple uORFs in the 5′UTR (41).

Note, however, that all previous studies on this topic haven’t directly analyzed measurements related to the SSU and haven’t quantified the effect of various features on the SSU at a genomic level. Thus, this is the topic of the current study.

Specifically, the aim of this study is to identify transcript features that affect the SSU scan based on direct measurement of the pre-initiation complex movement, to provide evidence for features that are considered to affect the scan and to find new undiscovered features, and to develop for the first time a computational predictive model for understanding translation initiation based on TCP-seq data. We identified different transcript features that affect the dynamics of the SSU scan, such as uAUG context score, uORF length, mRNA folding energy, various 5′UTR motifs etc., using quantitative tools such as linear regression, motif detection, and the Maximal Information Coefficient (MIC) (42). The results presented in this paper allow better modeling and engineering of initiation and the entire translation process.

## MATERIALS AND METHODS

### TCP-seq data

Translation Complex Profile Sequencing (TCP-seq) is a protocol that was developed from the Ribo-seq approach, designed to monitor the movement of the small subunit of the ribosome accompanied by initiation factors during the initiation step (26). The protocol was developed and implemented in *S. cerevisiae*, and can be found in (43).

In general, TCP-seq starts with snap-chilling of a yeast suspension culture by adding crushed ice and immediately cross-linking with formaldehyde, to stall and attach any translation complex type to the mRNA at their native positions. Next, RNase I was used to digest the unprotected mRNA fragments, generating protected (by any translation complex type) fragments, that will be referred as footprints. The ribosomal subunits, complete ribosomes and polyribosomes (polysomes) can be physically separated into different fractions by their sedimentation velocity, using two steps of density gradient ultracentrifugation: first, polysomes and mRNA associated with one complete ribosome, is concentrated into the pellet, whereas other messenger ribonucleoproteins, as well as free SSUs and LSUs, remain in the supernatant. Second ultracentrifugation is being used to separate the fractions of SSU and full ribosome complexes before retrieval of footprints, each associated with their respective protected mRNA footprints. Finally, the high-throughput sequencing reads were mapped to the yeast genome (43). The study was perform in YPAD in OD600 of 0.6–0.8 and not in stress conditions.

TCP-seq data provide the nucleotide footprints of the small subunit of the ribosome across the entire transcriptome of the analyzed organism at a single nucleotide resolution, as the footprint position is mapped by its 5′ end (see specific examples in Supplementary Figure S1). 5′UTR SSU footprints sizes ranged mainly from ∼15 to ∼75 nt (beyond the width of the SSU alone), due to the additional initiation factors that accompanies the SSU scan. It follows that each RC has a dependency both on SSU location in the 5′UTR and on footprint length.

### Sequencing data

TCP-seq footprint sequences were obtained from (26) (WT:Input, WT:RS and WT:SSU fractions, accession SRR3458591-3). Transcript sequences were obtained from Ensembl release 87 for S. cerevisiae (R64-1-1), with UTR annotations (longest isoform) from (44). Sequenced reads were mapped as described in (45) with the following minor modifications. We trimmed 3′ poly-A adaptors from the reads using Cutadapt (46) (version 1.17) and utilized Bowtie (47) (version 1.2.1) to map them to the *S. cerevisiae* transcriptome. In the first phase, we discarded reads that mapped to rRNA and tRNA sequences with Bowtie parameters ‘–n 2 –seedlen 21 –k 1 --norc’. In the second phase, we mapped the remaining reads to the transcriptome with Bowtie parameters ‘–v 2 –a --strata --best --norc –m 200’. We filtered out aligned reads >100 bp and <15 bp. First, unique alignments were assigned to the occupancy profiles. For multiple alignments, the best alignments in terms of number of mismatches were kept. Then, multiple aligned reads were distributed between locations according to the distribution of unique reads in the respective surrounding regions. To this end, a 100 nt window was used to compute the read count density RCD_*i*_ (total read counts in the window divided by length, based on unique reads) in vicinity of the *M* multiple aligned positions in the transcriptome, and the fraction of a read assigned to each position was ${{\rm RCD}{i}}/\sum_{j=1}^{M}{\rm RCD}_j$. The results are very similar when not including non-unique sequence.

### mRNA levels

For mRNA levels we considered measurements of RNA-seq from (48).

### Linear regression

Our aim was to predict the density of the SSU in sliding windows of 30 nucleotides (steps of 1 nucleotide), which is the size of the SSU alone. In order to predict the density of the SSU, we built a linear regressor based on the following scheme:

First, we generated a set of features that are related to various properties of the transcript (see sub-section ‘Features’). In total, we created a set of 399 transcript features. Next, the RC of the SSU in all footprint lengths were normalized by the mRNA levels of each gene. Out of a total number of 6664 genes of *S. cerevisiae*, 17 genes were excluded since their mRNA levels equal to zero and could not be normalized. All of the sliding windows were divided into three sets: train (60%), test (20%) and validation (20%), where the sampling was performed randomly 20 times, thus resulting in 20 predictors for each footprint length. This was done in order to avoid overfitting and to perform statistical analyses of the features that were selected by multiple predictors. We implemented a greedy feature selection process, meaning that in each iteration we added every feature to the growing regressor, and the feature that contributed the most to the correlation between the predictions and the real RC was selected. The process continues while the added feature raises the correlation by more 0.001, aiming to avoid overfitting to the train set. At the end of each stage, the current predicted regressor coefficients of the selected features are assessed on the test set. The selected regressor is then evaluated on the validation set. We chose to use Spearman correlation since it detects monotonic trends, while the Pearson correlation is strongly biased towards linear trends.

### Features

**Table:** 

	Feature	Feature description	Number of features
***Features derived from the genes***	The 5′UTR length		1
	The ratio of the length of the 5′UTR to the ORF		1
***Features derived from the sliding windows***	mRNA folding energy	Approximation of the mRNA secondary structure, calculated by MATLAB’s rnafold function	1
	AUG context score	A score for each AUG context (i.e. the nucleotides before and after the AUG).	1
	GC content	The percentage of nitrogenous bases on a DNA or RNA molecule that are either Guanine (G) or Cytosine (C).	1
	Nucleotide distribution	The frequency of each nucleotide in the sliding window	4
	Groups of two nucleotides distribution	The frequency of each nucleotide pair in the sliding window	16
	Groups of three nucleotides distribution	The frequency of each group of three nucleotides in the sliding window	64
	Sequence motifs	Short sub-sequences that are enriched in the 5′UTR.	293

### mRNA folding energy

The predicted mRNA folding energy is an approximation of the mRNA secondary structure, calculated by MATLAB’s rnafold function (MATLAB Bioinformatics Toolbox). The function predicts the folding energy of the secondary structure associated with the minimum free energy for an RNA sequence or subsequence in kcal/mol units.

We used a 30 nt sliding window in order to estimate the local mRNA folding energy, the same size as the sliding window in which we predicted the SSU density. We used this size for a number of reasons: first, this value is close to the size of the ribosome and the SSU, and in the order of magnitude of various intracellular complexes (2,49) and functional mRNA structures (34). Second, we aim at studying local mRNA folding, which is related to the local structures that are obtained on the mRNA when it starts to fold and before the folding is disrupted by various factors such as ribosomes. Third, we and others have shown that the results are robust to small changes in the size of the window (e.g. (50)). Last, the error of the RNA folding prediction tools is extremely high when working with windows larger than a few dozen nucleotides. Many others have used similar window size to predict the local mRNA folding energy (see, for example, (12,51–55)). See Supplementary Figure S2 for a dot plot which includes the actual folding energy for windows with different RC.

Several folding energy features were created for each sliding window: the folding energy in the current sliding window (position 1 in the sliding window); the folding energy in a sliding window within a distance of 30 nucleotides, which is the estimated size of the SSU (position 31); the mean and minimal folding energy in all sliding windows from the current sliding window to a sliding window within a distance of 30 nucleotides (positions 1:30); the mean and minimal folding energy in all sliding windows within a distance of 15/20 nucleotides from the current sliding window to a sliding window within a distance of 30 nucleotides (positions 15/20:30). See Figure 6A for an illustration of the above features.

### AUG context score

The score for each AUG context (i.e. the nucleotides before and after the AUG) was computed as follows for all AUGs in the 5′UTR: first, we selected the top 5% of highly expressed genes based on their mRNA levels. Second, we calculated a position specific scoring matrix (PSSM) for 6 nucleotides upstream and 3 nucleotides downstream to the initiating (START) AUG codon according to the nucleotide (A, C, G, U) appearance probability, based on the nucleotide context around the start codon of the selected highly expressed genes. Third, the AUG context score was computed for every AUG that appears in the 5′UTR according to:

$$\begin{equation*} AUG_{CS_j}=\sum {\rm log}(p_{ij}) \end{equation*}$$

Where j is the AUG index, i is the nucleotide position, *p_ij_* is the probability that the *i*th nucleotide of the *j*th AUG appears in the *i*th position on the PSSM, and *AUG_CS__j_* is the context score of the *j*th AUG in the 5′UTR.

For an AUG that appears in the sliding window without 6 nucleotides upstream or 3 nucleotides downstream (due to end-of-sequence positioning) we assumed a uniform distribution (i.e. a probability of 0.25 for each nucleotide). Several AUG-based features were created: the number of AUGs in the sliding window; a binary feature based on whether or not there is an AUG in the sliding window (‘1’- there are one or more AUGs in the sliding window, ‘0’- there isn’t an AUG in the sliding window); the mean distance of the AUGs in the sliding window from the real START AUG; the mean and maximal AUG context score in the sliding window; the mean and maximal AUG relative context score in the sliding window (the context score of the current AUG divided by the context score of the real START AUG of the ORF).

### Sequence motifs

Sequence motifs are short sub-sequences that are enriched in a certain set of sequences and are represented by Position-Specific Scoring Matrixes (PSSMs). To infer such PSSMs we used the HOMER (Hypergeometric Optimization of Motif EnRichment) tool (56). HOMER is based on a differential discovery algorithm which identifies sub-sequences that are specifically enriched in the target set relative to a reference set. We defined all of the 5′UTRs as the target set, and created the reference set in the following manner: for each 5′UTR we considered a sequence in the same length as the real UTR that is in a gap distance of 100 nucleotides upstream to the beginning of the real 5′UTR. The motivation was that since we are trying to find motifs specific to the 5′UTR that may have regulatory function, we’ll use a reference set that is *not* part of the UTR but close to it in the genome (thus, it may have similar mutation pattern and bias); this approach may allow us to detect motifs that are under selection in the 5′UTR due to their functionality. The length of the motif wasn’t pre-determined, the motifs were found using the algorithm from (56).

The algorithm detected 271 known motifs (all significant) and 32 de-novo motifs. Yet 6 of the de-novo motifs were marked as possible false discoveries and therefore were excluded from the features list. For each motif the HOMER algorithm outputs a PSSM; using that PSSM we calculated in each sliding window a set of scores for the motif according to the motif length. For example, if the length of the motif is 12 nucleotides, we moved it along the sliding window in positions 1:19 (out of 30), and for every position calculated the motif score. The score is calculated in the same way that was described above for the AUG context score. Finally, the feature that was set to the sliding window was the maximum score of all the values calculated in the window.

We have tried an additional approach for finding motifs as follows: we defined as target and reference sets windows with high and low SSU density (top and bottom 10% values, respectively); in order to avoid overfitting, this was done using only windows from the training group of each run. The motivation was that since we are trying to find motifs in the 5′UTR that may affect the SSU density, we’ll use the target and reference sets mentioned above, aiming to find motifs that are enriched in the high-density windows relative to low-density windows. The algorithm detected between 237 and 260 motifs (all significant), depending on the run number and the sliding windows that were used on the training set. These analyses gave very similar results to the results described in the article.

### Features analyses

In order to estimate how the features affect the dynamics of the SSU, we calculated the partial Spearman correlation between each feature and the RC of the SSU, while controlling for the rest of the features and mRNA levels.

Additional analyses were performed only on the folding energy features, allowing better estimation of the effect of folding energy strength on the SSU scan. First, we created an additional predictor that is based only on folding energy features, using 80% of the data to train the model. Next, we predicted the SSU density based only on these features, using 20% of the data as a test group, the same 20% that were used to evaluate the general predictor in order to ensure a fair comparison between them. Finally, we calculated the partial Spearman correlation between the predicted densities and the real RC, while controlling for the other features and mRNA levels.

### Detecting complex statistical relations between variables using MIC

In order to find how specific features affect the scanning of the SSU and compare between them, we used the Maximal Information Coefficient (MIC) (42), a measurement that is based on the mutual information of two variables. MIC enables detecting various relations (of any type) between pairs of variables in large data sets. Intuitively, the basic idea of MIC is that if we scatterplot two variables that have a relationship, then a grid can be drawn in a manner that will partition the data based on that relation. To calculate the MIC score related to the measurements of two-variable, we applied all grids in size of *x*-by-*y* to the data, up to a maximal resolution that is dependent on the sample size (42). The algorithm detects the grid with the maximal possible mutual information that can be achieved by any *x*-by-*y* grid. Then, the algorithm normalizes all of the mutual information values which allows for a fair comparison between different grid sizes and modified values between 0 and 1. The MIC is the maximal value of the characteristic matrix *M=(m_x,y_)*, which contains the highest normalized mutual information achieved by any *x*-by-*y* grid.

### Data processing

With the aim of finding which AUG features and sequence motifs affects the scanning of the SSU, we constructed a matrix for all AUGs or sequence motifs (according to the relevant analysis) in the 5′UTR that corresponds to a specific feature in the following manner: first, all of the relevant AUGs or sequence motifs in a given footprint length were aligned according to their first nucleotide position. Second, we looked on the RC of the SSU 50 nucleotides upstream and downstream to the relevant AUG or motif. Third, we normalized each position in the number of genes that have this location (since not all of the positions have 50 nucleotides upstream). The above was done for all footprint lengths (15–100 nucleotides), resulting in a matrix of all the relevant AUGs or sequence motifs corresponding to the specific feature, depending on both location in 5′UTR and footprint length.

### Discretization process

In order to use the MIC, a discretization process needs to be performed due to the dependency on both location and footprint length, meaning that we transferred a grey scale matrix into a binary one. Several discretization processes were tested. First, a discretization process that sets a threshold for each row (i.e. footprint length) as the threshold that maximizes the MIC score of the entire matrix. Second, in order to show that the results stay robust and that they are not a result of the optimization process, manually selected parameters were set as the threshold for each row. The thresholds that we tested are mean *(m)*, 2*m, mean plus standard deviation, and median.

### Null model and *P* value

For each signal we created 1000 null model randomizations by permutating the matrix of the constructed signal along the *x* axis (location relative to AUG or sequence motif in the 5′UTR). For every permutated matrix, the discretization process described above was performed and the MIC score was computed. The *P* value was calculated as the number of times that the randomized matrix had a higher MIC score than the real one, divided by the total number of permutations.

An additional permutation process was tested; in order to show that the results stay robust and that they don’t stem from the number points that passes the threshold, we created 1000 additional permutations for each signal after the discretization process was done. For each signal, we controlled the number of points that passes the threshold by using the next iterative process: we started by adding epsilon = 1 × 10^−5^ to the threshold of each row. If the number of points that passes the threshold was still too large—the process continues, and epsilon is added again. When we reached the desired number of points—the process stopped. For each comparison between signals, the number of points that passes the threshold was aligned to the signal with the lowest number of points.

### AUGs in 5′UTR with high/low context score

First, we computed the AUG context score for all AUGs in 5′UTR in the same way as described above (see sub-section ‘AUG context score’ under ‘Features’). Next, we divided them into two groups of high and low context score, according to top and bottom 10% scores, respectively. The ‘high context score’ group contained 2587 AUGs, and the ‘low context score’ group contained 2333 AUGs. In order to compare between them and the real AUG START codon, we randomly chose 2465 genes out of 6664 genes of *S. cerevisiae* for analyzing the signal surrounding the main AUG START codon. The results stay robust to different group of genes that were selected. For each group we constructed the signal as was described above, then the MIC score and the *P* value were calculated.

We performed the same analysis for AUG-like codons, using the same PSSM which was used for AUG codon, to calculate the MIC score around AUG-like codons (CUG, AUU, AGG). For comparison we also analyzed all the rest of the codons. In each case, we considered both the MIC score surrounding the codon and the actual pattern of RC accumulation upstream of the codon.

### AUGs in 5′UTR with short/long distance to the nearest STOP codon

The uORF that we analyzed were simply define by us as a sequence of nucleotides starting with AUG ‘codon’/triplet, ending with a ‘stop’/triplet and divided by three. We did not consider in addition an experimental evidence as we wanted to get a general result which are not affect by experimental bias and/or are partial. In order to examine the effect of the distance from the nearest STOP codon (‘UAA’, ‘UAG’, ‘UGA’), we divided the AUGs to two groups of long and short distance from the nearest STOP codon, according to the top and bottom 20% distances, respectively. For these analyses we considered three optional reading frames: frame 0 is the reading frame that correspond to the relevant AUG, frames 1 and 2 represent a frame shift of 1 or 2 nucleotides relative to frame 0. For each group in each frame we constructed the signal as was described above. Then the MIC score and the empirical *P* value were calculated.

The last two sub-sections were performed twice; first to consider all AUGs that correspond to a specific feature, and again to take in consideration only AUGs that don’t have an additional AUG codon in the sequence 50 nucleotides downstream or upstream to the relevant AUG.

### AUGs in 5′UTR with presence of stable/unstable structure downstream

In order to examine the effect of the presence of stable structures downstream to the uAUG, we first defined stable/unstable structure as 10% lowest/highest folding energy values, respectively. We considered uAUGs with high/low folding energy on average in the 20 sliding windows downstream to the AUG, as only one position is not necessarily indicative for definition of stable/unstable structure. For each group we constructed the signal as was described above, then the MIC score and the *P* value were calculated.

We also tested the signal for short and long uORFs. In this case, we changed the cut-offs of stable/unstable structure to 50% lowest/highest folding energy values, respectively. This is because using a 10% threshold resulted very small number of relevant uAUGs, which might lead to biased and unreliable results. For a higher cut-off, we obtained similar number (as in previous signals) of uAUGs.

### Motifs in the 5′UTR with high/low score

The objective of this section was to apply the MIC calculations on sequence motifs that were selected many times by the predictors in order to examine how they affect the SSU scan. First, for each motif, we calculated the motif score in sliding window in size of the motif along the 5′UTR (see sub-section ‘HOMER motifs’ under ‘Features’). Next, we divided the windows into two groups of high and low score, according to top and bottom 5% scores, respectively. The groups contained a similar number of motif windows that constructed the signal, about 88,000 in size. Finally, for each group we constructed the signal as was described above. Then the MIC score and the *P* value were calculated.

## RESULTS

In order to understand the dynamic of the SSU scan from the 5′ to the 3′ of the mRNA, we analyzed TCP-seq data, a protocol that provides the footprints of the SSU of the ribosome across the entire transcriptome at a resolution of single nucleotide (26) (see Materials and methods section). The scan, which begins in the 5′cap and moves forward in the 3′ direction until the detection of the AUG start codon, is accompanied by different initiation factors (5,57), resulting in footprints that have dependency both on the location of the SSU in the 5′UTR and on footprint length (Figure 1A). A total number of 482,390 RC were mapped onto 6664 genes of *S. cerevisiae*. SSU footprints sizes ranged mainly from ∼15 to ∼75 nt (Figure 1B).

As can be seen in Figure 1B,C and previously published in (26), the SSU RC coalesced into three major sizes (19, 29 and 37 nt) at the main AUG start codons. During initiation phase, the SSU scans the 5′UTR until the detection of the start codon and is joined by the large subunit of the ribosome to form the 80S complete ribosome. For that reason, the highest amount of RC observed is prior to the main AUG start codon (as can be seen if Figure 1C). The start-codon-associated SSU footprints exhibited three main lengths, 19, 29 and 37 nucleotides, mainly due to dynamic rearrangements at the entry to the mRNA channel- from an open to a closed state following start codon recognition (26). FP length of 19 nucleotides probably derive from SSU paused at the start codon, yet it is still in an open, scanning-component configuration. Next, the SSU moves to a close state, protecting 29 nucleotides, following the recognition of the start codon. Finally, the third state corresponds to a state where eIF5B:GTP attaches the entry of the mRNA channel just before the large subunit of the ribosome joins. As a result, the protection is further extended to 37 nucleotides. An illustration of the process can be seen in Figure 1D.

In the following research we tested two different, yet potentially complementary, approaches, in order to study the dynamics of the SSU scan.

The first approach was based on linear regression, in order to meet two main objectives. The first one was to predict the density of the SSU based on transcript features and compare the results to the SSU RC from the TCP-seq data. The second objective was to perform comprehensive feature analyses, aiming to reveal how different transcript features affect the scanning of the SSU.

**Figure 2.**
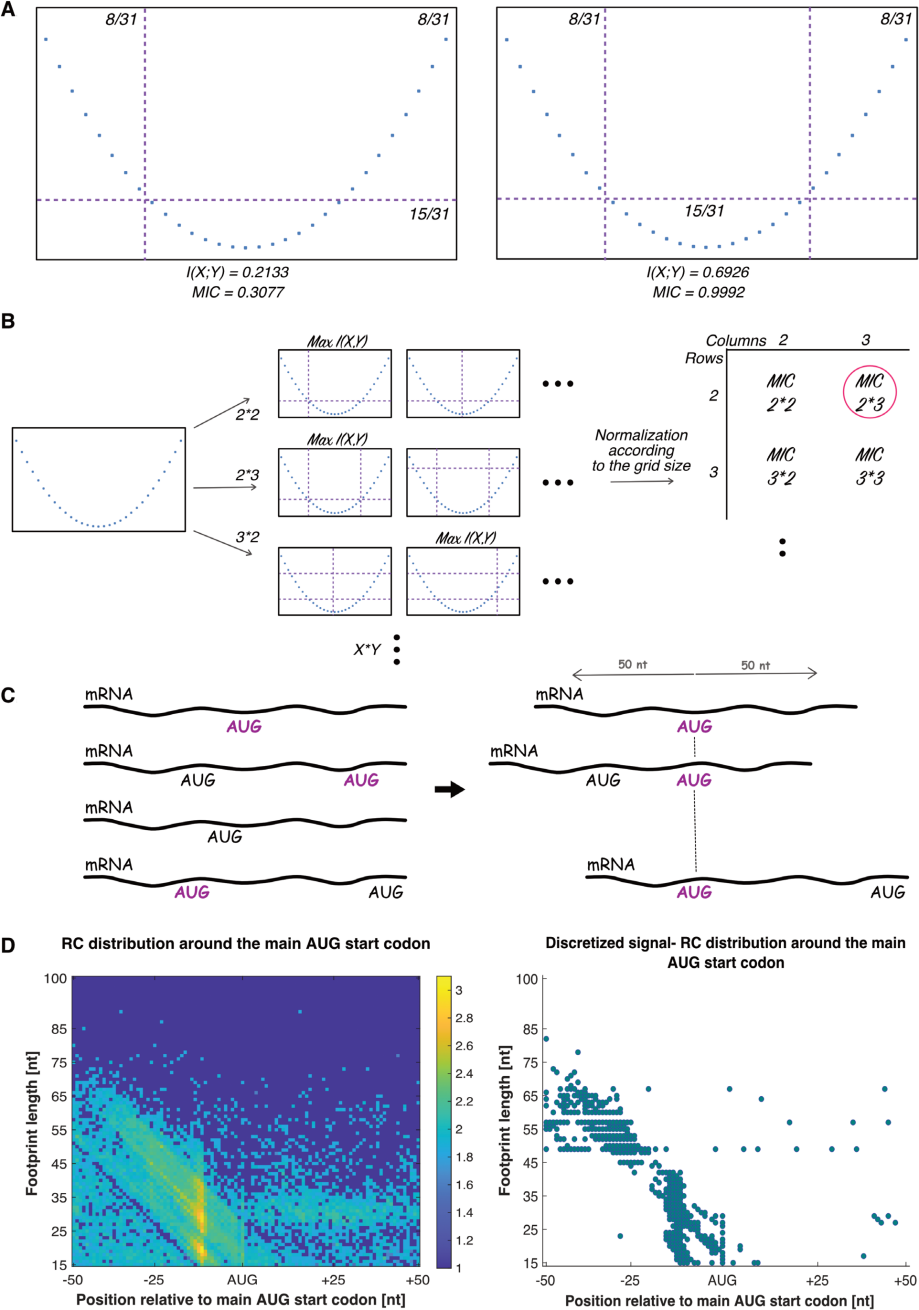
Illustration of MIC computation and its application on TCP-seq data. (**A**). An example of simple association between two variables with 15 data points. Two different grids are drawn, a 2-by-2 and a 2-by-3 grids. Using the probability to see data points in each bin, we calculated the resulted mutual information I(X,Y) for each grid. The 2-by-3 grid resulted in a higher mutual information compared to the 2-by-2 grid. (**B**) The MIC algorithm searches for the X-by-Y grid that maximizes the mutual information of the two variables for each pair (X,Y). It then compiles it to a matrix that stores the best score at that resolution, where the scores are normalized in order to compare between the different resolutions. The MIC corresponds to the maximal value of the matrix (circled in pink) (42). More details in the Materials and methods section. (**C**) For each AUG and sequence motif in the 5′UTR, we considered related features that surround it. For example, AUGs corresponding to a specific feature are marked in purple. We analyzed all of the AUGs with a specific feature by aligning their RC profiles according to the AUG position. (**D**) An example for resulted matrix of the SSU RC distribution surrounding AUGs with a specific feature (RC distribution surrounding the main AUG start codon), before and after discretization. The MIC algorithm was applied on the discretized image on the right and resulted in a high MIC score of 0.8795.

The second approach was based on the Maximal Information Coefficient (MIC) (42), tailored to the TCP-seq data. We named it Translation Complex Profile Information Coefficient (TCP-IC), and it was used in order to detect complex statistical relations between variables, which allowed us to perform computational analyses to examine the effect of different features on the SSU scan. The motivation to use MIC is that it enables detecting various relations (of any type) between pairs of variables in large data sets and can be used for analyzing the distribution of all the read lengths together. Intuitively, MIC estimates the mutual information between two variables based on a scatterplot of the two variables (Figure 2A,B). MIC score X is analogous to a correlation of *R*^2^ = *X* (e.g. MIC score of 0.8795 it is equivalent to *R* = 0.9378) (42,58).

In our case, for each AUG or sequence motif in the 5′UTR that was examined, we considered related features that surround it. Thus, we analyzed AUGs and motifs with specific features near it via the alignment of all of the RC profiles surrounding these relevant AUGs and motifs, creating a matrix of the SSU RC surrounding the relevant position (Figure 2C,D). Due to the dependency of the RC on both location in the 5′UTR and on footprint length, the data were discretized (i.e. it was transferred from a grey scale image to a binary one) in order to use the MIC (Figures 2D and 8). Then, for each matrix we calculated the MIC score and *P* value in order to compare between the different features.

Using these two approaches on the TCP-seq data allowed us to perform wide analyses and to get a broader understanding of the SSU scan during the initiation phase.

### Prediction of the small subunit density based on transcript features

In the following section, we used linear regression in order to predict the SSU density based on transcript features alone, as we trained and tested the model using the RC from the TCP-seq data. In general, we divided all of the genes into sliding windows in size of 30 nucleotides, which is the size of the SSU alone. For each sliding window we calculated a set of features, including the 5′UTR length, different features related to the mRNA folding strength, GC content, uAUG related features, the frequency of nucleotides, pairs of nucleotides and triplets of nucleotides, motifs (sub-sequences) in the 5′UTR, etc. (see ‘Features’ in the Materials and methods section). In total, we created a set of 399 transcript features. All of the sliding windows and densities vectors were divided into three sets: train (60%), test (20%) and validation (20%), where the sampling was performed randomly 20 times, thus resulting 20 predictors in each footprint length. This was done in order to avoid overfitting and to perform statistical analyses of the features that were selected by multiple predictors. We trained the model on the training set and assessed it on the test set, where in each iteration we added the feature that contributed the most to the correlation between the predictions and the real RC (greedy feature selection process). An independent model was trained with a different set of optimized features for each footprint length. Finally, for each predictor we calculated the Spearman correlation between the predicted RC and the real RC.

**Figure 3.**
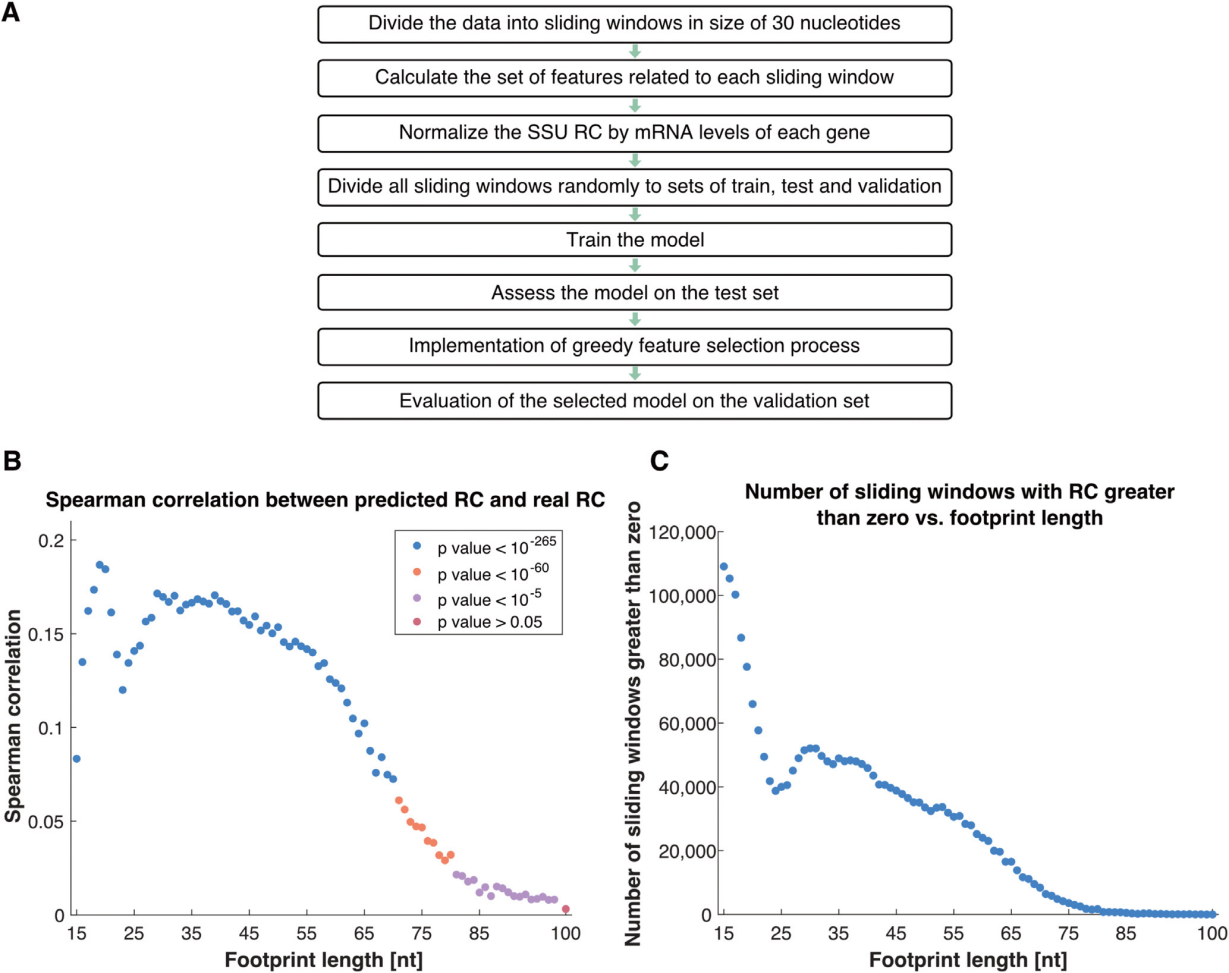
Prediction of the SSU density based on transcript features. (**A**) The steps in producing the predictors. The division to groups of train, test and validation was performed randomly 20 times, resulting in 20 predictors for each footprint length. (**B**) Spearman correlation between the predicted RC and the real RC, as function of footprint length. The presented values are the median correlations obtained from all 20 predictors in each footprint length, and the colors corresponds to the *P* values. (**C**) Number of sliding windows (out of total number of 1,626,494 sliding windows) that have real RC greater than zero as function of footprint length.

The obtained Spearman correlations between the predicted RC and the real RC are relatively low, probably due to various sources of bias and noise in the data and the very large number of points, yet very significant, as the *P* values are extremely low for most of the footprint lengths (Figure 3B). The *P* values remain significant for Pearson correlation as well (Supplementary Figure S3). The correlations decrease for longer footprint lengths, a trend that can be explained by the number of sliding windows with densities greater than zero (Figure 3C). For longer footprint lengths there are fewer sliding windows with densities greater than zero, meaning the model doesn’t have enough data to learn from.

**Figure 4.**
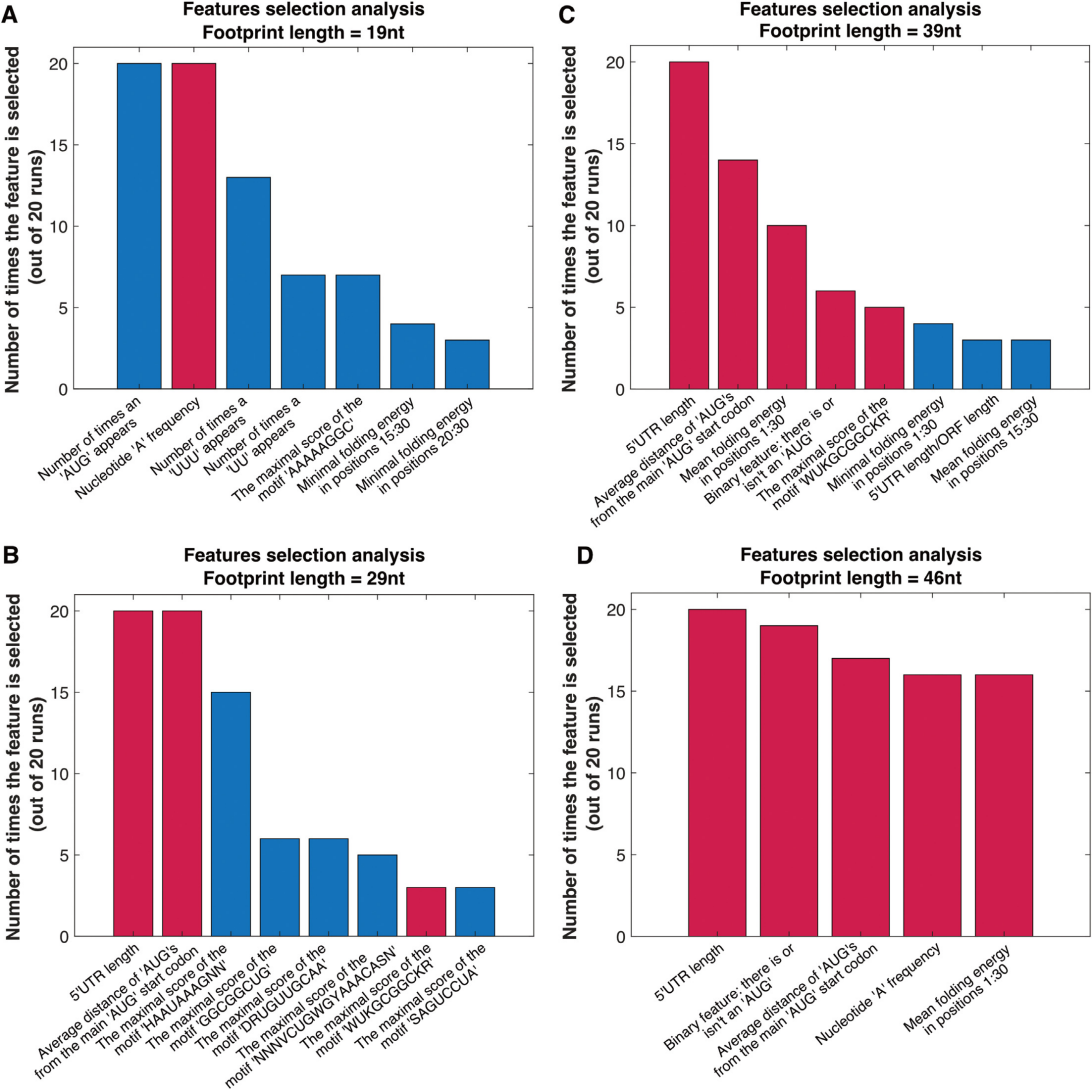
Feature selection analyses. The top features according to the number of predictors they participated in, for footprint lengths of (**A**) 19 nucleotides, (**B**) 29 nucleotides, (**C**) 39 nucleotides and (**D**) 46 nucleotides. Bars colored in red correspond to features that appear in more than one footprint length, and bars colored in blue correspond to features that appear only for one length.

The first statistical analysis we performed was to examine the distribution of the features selected in footprint lengths that yielded relatively high correlations between the real RC and the predicted RC. We obtained four local maximum points in Figure 3B (of four different footprint length regions: 15–24 nucleotides, 25–34 nucleotides, 35–44 nucleotides and >45 nucleotides) and ranked the features according to their number of appearances in the regressors generated via the sampling mentioned above. The maximal value is 20, as the number of predictors built for each footprint length (Figure 4).

Different footprint lengths produce different feature selection distributions; however, some features were prominent in all the distributions examined: the 5′UTR length; features related to uAUGs, including a binary feature that describes whether there is or isn’t an AUG in the current sliding window and the average distance from the main AUG START codon, and features related to the folding strength. The predictors also included several different novel motifs in the different footprint lengths; a comprehensive analysis of these motifs appears later in the paper.

The second statistical analysis we performed was to examine the prevalence of the top features used in many of the predictors for all footprint lengths (Supplementary Figure S4). The features were ranked according to their total number of appearances in regressors (see full table in Supplementary Table S1). Next, to understand the effect of the features on the SSU density, we calculated the partial Spearman correlation between each feature and the real RC, while controlling for the other features and the mRNA levels. Note that we have performed additional analyses based only on the folding strength features (appears next), therefore the results relevant to these features will not be presented in this section (we have included them in the analyses, the aforementioned is only regarding the presentation of the results). Overall, from all of these analyses we studied the relation between each feature and the SSU density: what is the importance of the feature, in which directionality it affects the density, in which footprint lengths the partial correlation is significant etc.

**Figure 5.**
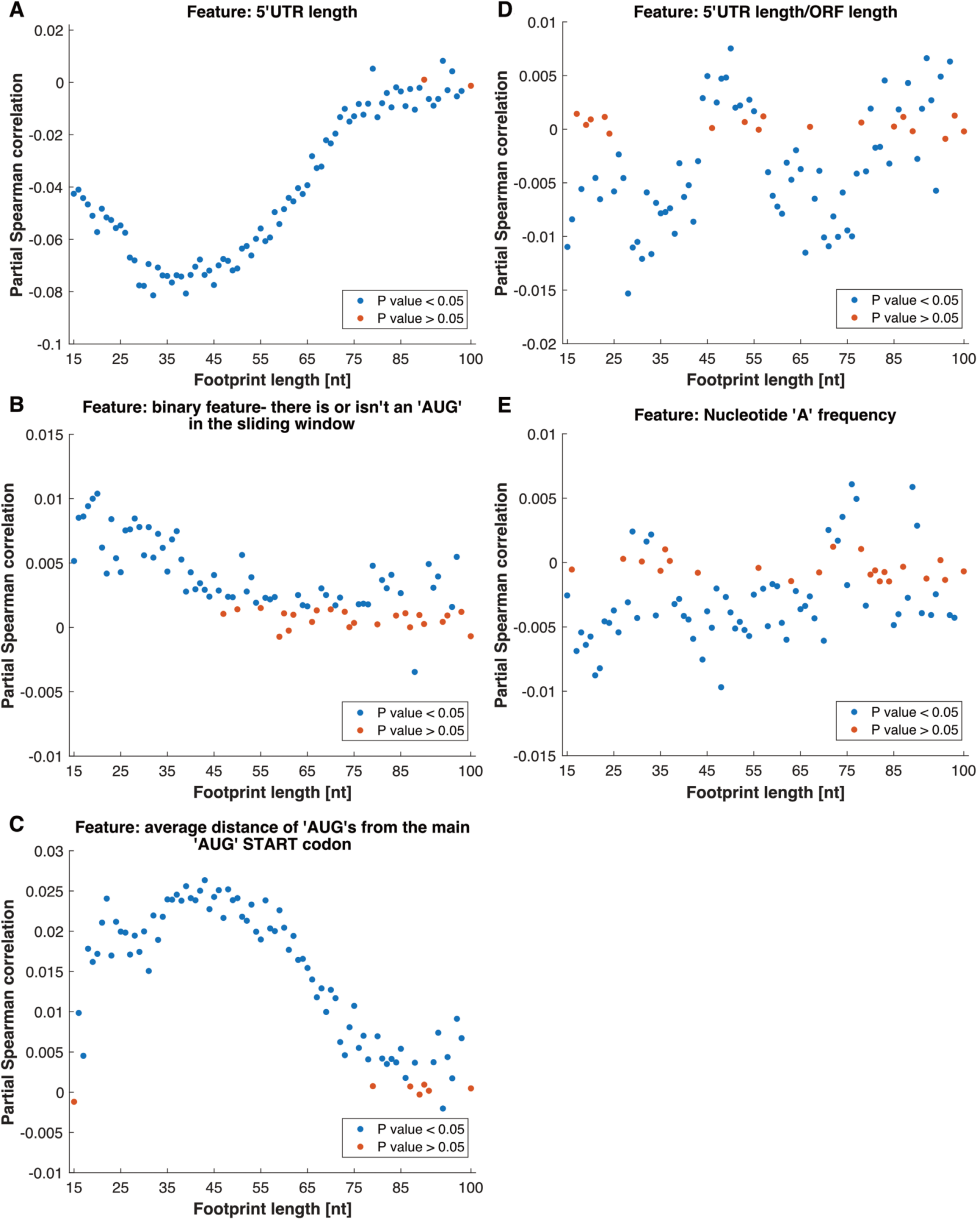
Partial correlation of features selected by multiple predictors with the SSU read counts. Partial Spearman correlation between each feature and the SSU RC, while controlling for the other features and the mRNA levels.

The feature that was selected most frequently is the 5′UTR length and its partial correlation analysis shows an inverse relation to RC, as shorter UTRs tend to have more RC (Figure 5A). Also at the top of the ranking are features related to uAUG. One is a binary feature that describes whether there is or isn’t an AUG in the current sliding window, where the partial correlations of the feature with the RC are mostly positive (Figure 5B), meaning that if there is an AUG in the sliding window there are more RC. The average distance to the main AUG START codon was also selected multiple times, and from the partial correlation analysis we see that for longer distance from the main AUG there are more RC (Figure 5C). In addition, the 5′UTR length relative to the ORF length was also selected frequently, presenting mostly negative partial correlations with respect to RC (Figure 5D). Other top selected features are: features related to the folding strength of the mRNA (see next section); nucleotide ‘A’ frequency (Figure 5E); the number of ‘AUG’s in the sliding window, GC content and the maximal AUG context score in the current sliding window (Supplementary Figure S5).

### Strong mRNA folding in the 5′UTR contributes to the abortion of the small subunit

**Figure 6.**
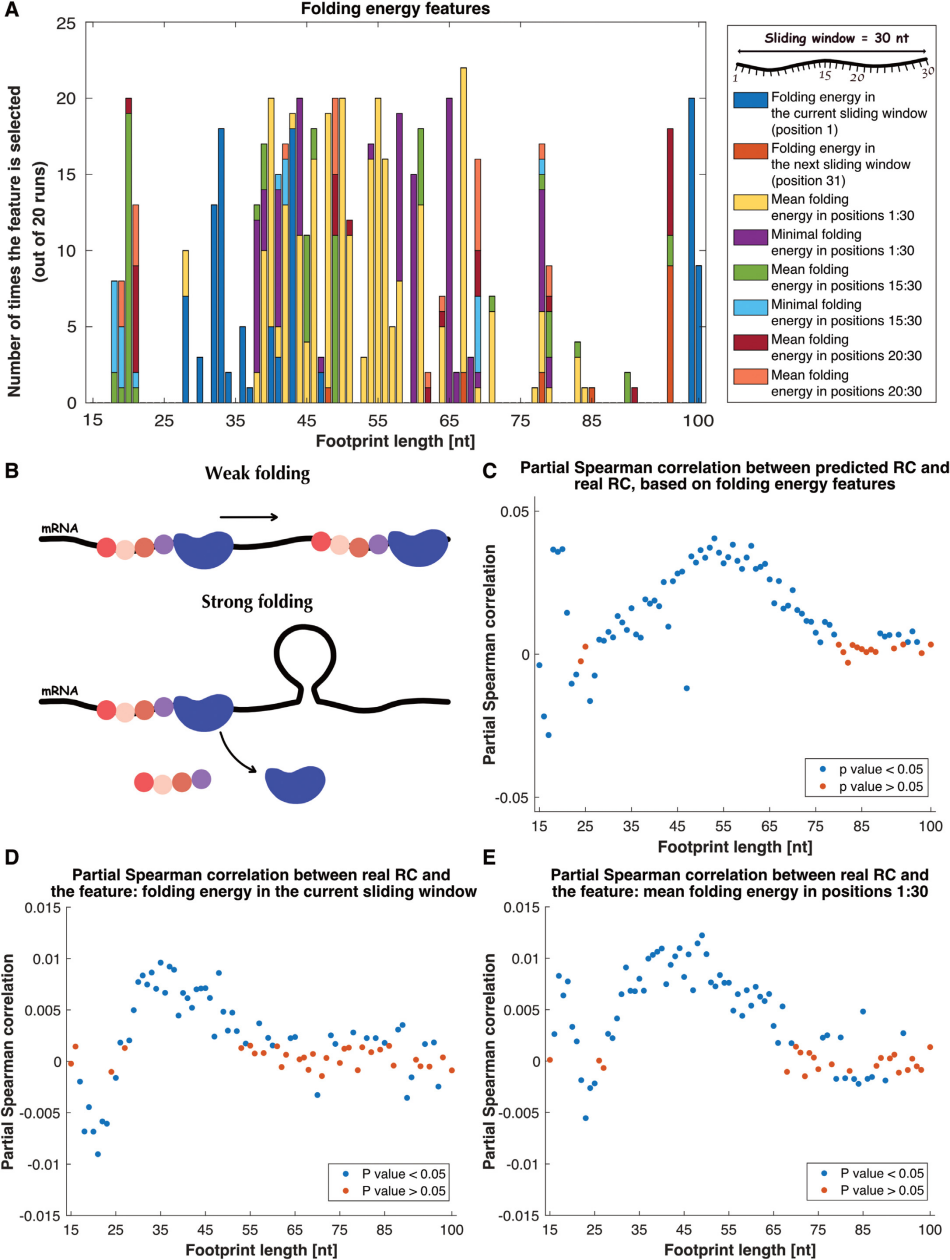
Folding strength analyses. (**A**) Number of times that features related to the folding energy were selected, as function of footprint length. (**B**) Illustration of weak folding (i.e. high folding energy) and strong folding (i.e. low folding energy). (**C**) Prediction of the SSU density using only folding energy features. The partial Spearman correlation between the real RC and the predicted RC, while controlling for the other features and the mRNA levels. The presented values are the median correlations obtained from all 20 predictors in each footprint length, and the colors corresponds to the *P* values. (**D**), (**E**) Partial Spearman correlation between folding energy features and the real RC, while controlling for the other features and mRNA levels, as function of footprint length.

Since the effect of the local folding strength of the mRNA is not trivial for detection by only one feature, we created set of features related to the folding energy of the mRNA, including the folding energy in the current sliding window; the folding energy in the next sliding window (since we expect to see an influence of the next position on the current position, regarding the density of the SSU), and the mean and minimal folding energy values in different positions along the sliding window (Figure 6A). When we examined the prevalence of the aforementioned features, we saw that they were selected multiple times by all of the predictors in all footprint lengths (Figure 6A). Therefore, we performed additional analyses using all of the folding energy features; we created additional predictors, this time based only on the folding energy features, while using the same indices that were used in the main predictors to train and validate the model in order to allow a fair comparison between the analyses.

The folding energy and the folding strength are inversely related; higher folding energy means a weaker folding strength and vice versa (Figure 6B).

When calculating the partial Spearman correlations between the predicted RC and the real RC, controlling for the other features and the mRNA levels, the observed correlations were mainly positive and significant (Figure 6C). Moreover, the examination of all partial correlations between each feature and the RC yielded mostly positive partial correlation (e.g. Figure 6D,E, see all figures in Supplementary Figures S6, S7), meaning that for less negative folding energy (i.e. weaker folding) there are more RC. This result is different from what we have seen in the cases of the complete ribosomes, where stronger folding tend to slow down the ribosome and thus increase its density (see, for example (21,59)). A possible biophysical explanation is that when the SSU reaches a strongly folded area, since it has only a limited ability to disrupt complicated secondary structures relative to the entire ribosome during the elongation phase (38,39), it is possible that it detaches from the mRNA. The analysis of the GC content feature reinforces the claim, as the partial correlations between the feature and the RC are negative, meaning that for higher GC content (i.e. a more stable structure) there are less RC (Supplementary Figure S5).

### Higher context score and uORF length induce RC distribution more similar to the RC surrounding the main AUG

In the current and following sub-sections we performed a higher resolution analysis of some of the transcript features that affect the SSU movement using MIC, introducing a new method tailored for the TCP-seq data: the Translation Complex Profile Information Coefficient (TCP-IC).

With the aim of finding which start codon-associated features affect the scanning of the SSU and the strength of their effect, we constructed a matrix for all AUGs in the 5′UTR that are related to each specific feature. The first feature that was tested is the context (i.e. the nucleotides composition before and after the AUG) score of all AUGs in the 5′UTR, a score that is based on the comparison to the nucleotide distribution around the main AUG at the beginning of the ORF of the most highly translated genes (60). For highly translated genes we calculated a position specific scoring matrix for 6 nucleotides upstream and 3 nucleotides downstream to the main AUG start codon according to the relative frequency of nucleotides, and based on it we computed a score to each AUG in the 5′UTR (see Materials and methods section). As was reported in a previous study (60), the correlation between the optimality of the context score and the protein levels was relatively low but significant. Based on these scores, we examined how high and low context scores (top and bottom 10% values, respectively) affect the behavior of the SSU, using the MIC analyses.

**Figure 7.**
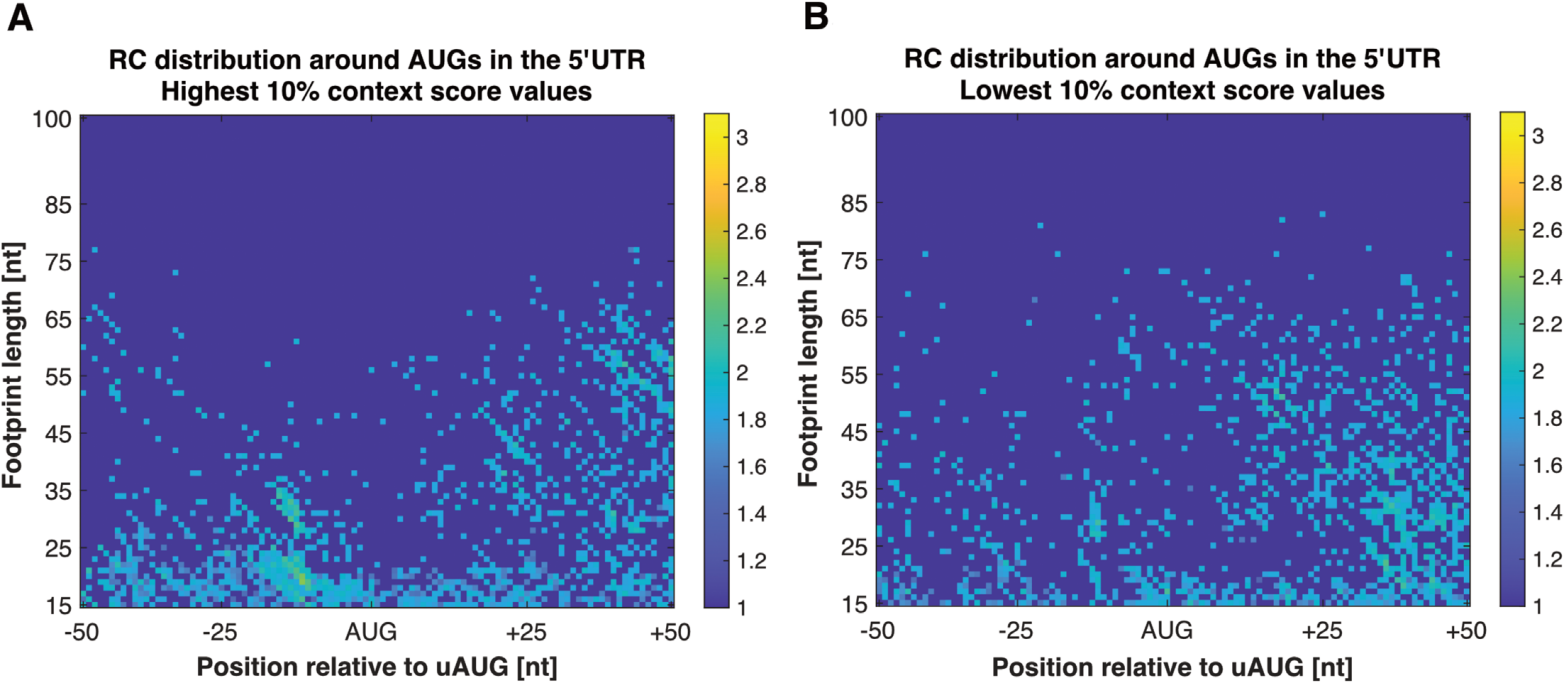
RC distribution surrounding AUGs in the 5′UTR with high/ low AUG context score. (**A**) RC distribution around AUGs in the 5′UTR with high AUG context score. It can be seen that AUGs with high context score present a trend that is more similar to the RC distribution surrounding the main AUG start codon. (**B**) RC distribution around AUGs in the 5′UTR with low AUG context score. No trend was identified, neither by the scatterplot or by the MIC score analyses. The results stay robust to different selections of genes.

The results show that AUGs in the 5′UTR with high context score have a higher MIC score (0.5230) relative to AUGs with low context score (0.1389), yet lower than the MIC score of the main AUG start codon (0.8795) (Table 1). The pattern of the RC accumulation can be seen in Figure 7 and Supplementary Figure S8. The results stay robust to different discretization thresholds that were tested, and to the examination of the signal with the constrain that the relevant AUG does not have another AUG in the region 50 nucleotides upstream or downstream (Supplementary Table S3). The reported results indicate that AUGs with high context score induce relatively similar RC distribution to the RC surrounding the main AUG start codon, meaning it affects the scan of the SSU by making it linger around these AUGs. Our analysis suggest that AUG context affects the small sub-unit movement and contribute to ∼50% of the variance related to the RC distribution near the main start codon and over 17% near UTR AUGs with high context score. The conclusions remain similar for highly expressed genes (Supplementary Figure S9).

**Table 1.** AUGs in the 5′UTR with higher context score induce RC distribution similar to the RC surrounding the main AUG

	MIC score	*P* value	Total Number of AUGs that constructed the signal	Number of points that passes the threshold
Main AUG start codon	0.8795	<0.001	496	439
AUGs with high AUG context score	0.5230	<0.001	504	431
AUGs with low AUG context score	0.1389	0.08	446	434

AUGs in the 5′UTR with higher context score has a higher MIC score, similar to the MIC score of the main AUG start codon. The number of AUGs that constructed the signal (see Materials and methods section) and number of points that passes the threshold were controlled and aligned to all signals. Number of points passes the threshold refers to a control process we performed, aiming to show that the obtained MIC score is not a result of different number of points remaining after the discretization process was completed. Further details can be found in the Materials and methods section. MIC score and *P* value were calculated as described in the Materials and methods section. The results presented in the table are for AUGs that don’t have additional AUG 50 nucleotides upstream and downstream, while using discretization process that sets the threshold of each row (i.e. footprint length) as the threshold that optimizes the MIC score of the entire matrix. The results stay robust to all thresholds that were tested.

This is the first large scale *direct* quantitative evidence showing that AUG context does in fact affects the small sub-unit movement.

We performed the same analysis for AUGs in the 5′UTR as function of their distance from the nearest downstream STOP codon in order to examine how the uORF length affects the scanning of the SSU and related to signals that modulate this scanning. We divided all of the relevant AUGs into two groups: those that are either a long or short distance from the nearest STOP codon. We used the top and bottom 20% of distances, respectively. The analyses were performed in all three optional reading frames (Frame 0, Frame 1, and Frame 2 relative to the AUG in the 5′UTR). The results show that in frame 0, AUGs that are farther away from the nearest STOP codon, tend to have a higher MIC score relative to AUGs that are closer (0.2838 compared to 0.1990, respectively). These results indicate that longer uORFs (longer than 87 nucleotides) tend to be associated with RC distribution that is more similar to the RC surrounding the main AUG and thus probably tend to trigger initiation. Similar results were obtained when we analyzed only highly expressed genes. In frames 1 and 2 we did not observe the same trend as in frame 0, possibly because in frame 0 there are additional/stronger signals surrounding the AUG that are playing a role in triggering translation.

**Figure 8.**
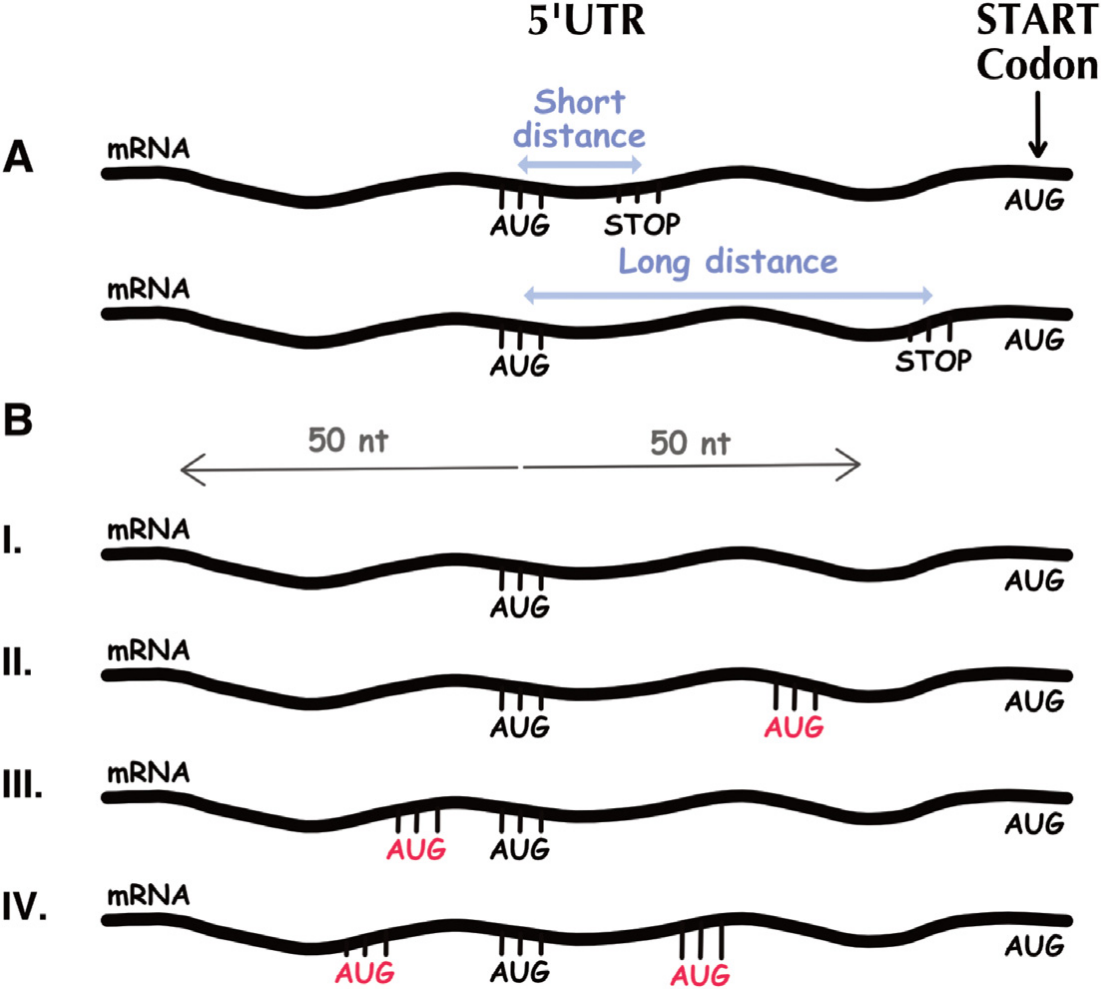
Illustration of uORF length analyses. (**A**) AUGs in the 5′UTR with short/long distance from the nearest STOP codon (‘UAA’, ‘UAG’, ‘UGA’). Short distance is defined as about 20% of the lower distance values, e.g., in frame 0, all AUGs that their distance from the nearest STOP codon is <9 nucleotides. Long distance is defined as 20% of the upper distance values. In frame 0 long distance corresponds to all AUGs that their distance from the nearest STOP codon is >87 nucleotides. (**B**) Further analyses have been done in frame 0 to test the influence of additional AUG in the surrounding of 50 nucleotides downstream and upstream to the examined AUG. We divided the groups mentioned above (short/long distance) into 4 sub-groups: (I) AUGs that don’t have additional AUG downstream or upstream, (II) AUGs that have additional AUG downstream, (III) AUGs that have additional AUG upstream and (IV) AUGs that have additional AUG both downstream and upstream.

Further research has been done only for frame 0, as we performed the same analysis as above, this time in order to test the influence of additional AUG nearby. All of the AUGs in the 5′UTR that corresponds to a short or long uORF were further divided into 4 sub-groups (total number of 8 groups): (i) AUGs without any additional AUG within 50 nucleotides upstream and downstream, (ii) AUGs with additional AUG within 50 nucleotides downstream, (iii) AUGs with additional AUG within 50 nucleotides upstream and (iv) AUGs with additional AUG within 50 nucleotides both upstream and downstream (group numbers correspond to Figure 8B (I)–(IV)). In most of the cases that were tested for AUGs with short distance to the nearest STOP codon, additional AUG upstream decreases the MIC score of the signal, in comparison to the case that there isn’t any additional AUG in the surrounding sequence (Table 2). This result supports the scanning model of translation initiation (28). However, for AUGs far from the nearest STOP codon we see a reverse relationship; an upstream AUG is related to a higher MIC score, probably due to additional encoded signals in this case.

**Table 2.** The effect of additional AUG in the surrounding of short and long uORFs

	MIC score	*P* value	Total Number of AUGs that constructed the signal	Number of points that passes the threshold
**Short uORF**				
(I) Without another AUG	0.1990	<0.001	762	525
(II) Additional AUG downstream	0.2373	<0.001	762	587
(III) Additional AUG upstream	0.1763	<0.001	762	578
(IV) Additional AUG upstream & downstream	0.2410	<0.001	762	594
**Long uORF**				
(I) Without another AUG	0.2838	<0.001	881	404
(II) Additional AUG downstream	0.1690	0.02	881	361
(III) Additional AUG upstream	0.3051	<0.001	881	456
(IV) Additional AUG upstream & downstream	0.4008	<0.001	881	379

Group numbers correspond to Figure 8B (I)–(IV). For short uORFs in the 5′UTR, additional AUG upstream decreases the MIC score of the signal in comparison to the case where there isn’t any additional AUG in the surrounding. For long uORFs the relation is reversed; an upstream AUG corresponds to higher MIC score, possibly due to additional signals encoded in this case. For both parts of the table the number of AUGs that constructed the signal and number of points that passes the threshold were controlled and aligned to all signals. Number of points passes the threshold refers to a control process we performed, aiming to show that the obtained MIC score is not a result of different number of points remaining after the discretization process was completed. Further details can be found in the Materials and methods section. MIC score and *P* value were calculated as described in the Materials and methods section. The results presented in the table are for using discretization process that sets the threshold of each row (i.e. footprint length) as the threshold that optimizes the MIC score of the entire matrix.

For short uORFs, additional AUGs downstream actually increases the score of the signal, possibly since these AUG codons tend to be non-functional and the additional AUG may be more functional. Furthermore, for both short and long uORFs in the 5′UTR, the case of additional AUG both upstream and downstream resulted in the highest MIC score (for each part of the table, respectively); a possible explanation is that in this case, due to the presence of several AUGs, there is a higher RC in the surrounding area and as a result a higher MIC score is obtained.

### Estimating the effect of non-AUG codon on SSU RC

In order to test the influence of AUG-like codons on the SSU RC, we performed the same analyses for ‘CUG’, ‘AUU’ and ‘AGG’ codons. We used the same PSSM which was used for the AUG codon analyses. For comparison we also analyzed all the rest of the codons. In each case we considered both the MIC score surrounding the codon and the actual pattern of RC accumulation upstream of the codon.

We found that AUG has the top MIC score in comparison to all other codons. Some of the AUG-like codons have relatively high MIC score and RC accumulation upstream of the codon. However, we found additional codons with high MIC and RC accumulation upstream of the codon, such as CAG. Nevertheless, the difference between CAG with high AUG context score and CAG with low AUG context score is smaller compared to the AUG codon. Additional information can be found in Supplementary results and Supplementary Figures S10, S11 and Supplementary Tables S4 and S5.

### Presence of stable/unstable structure downstream to uAUGs affect RC and MIC score

**Figure 9.**
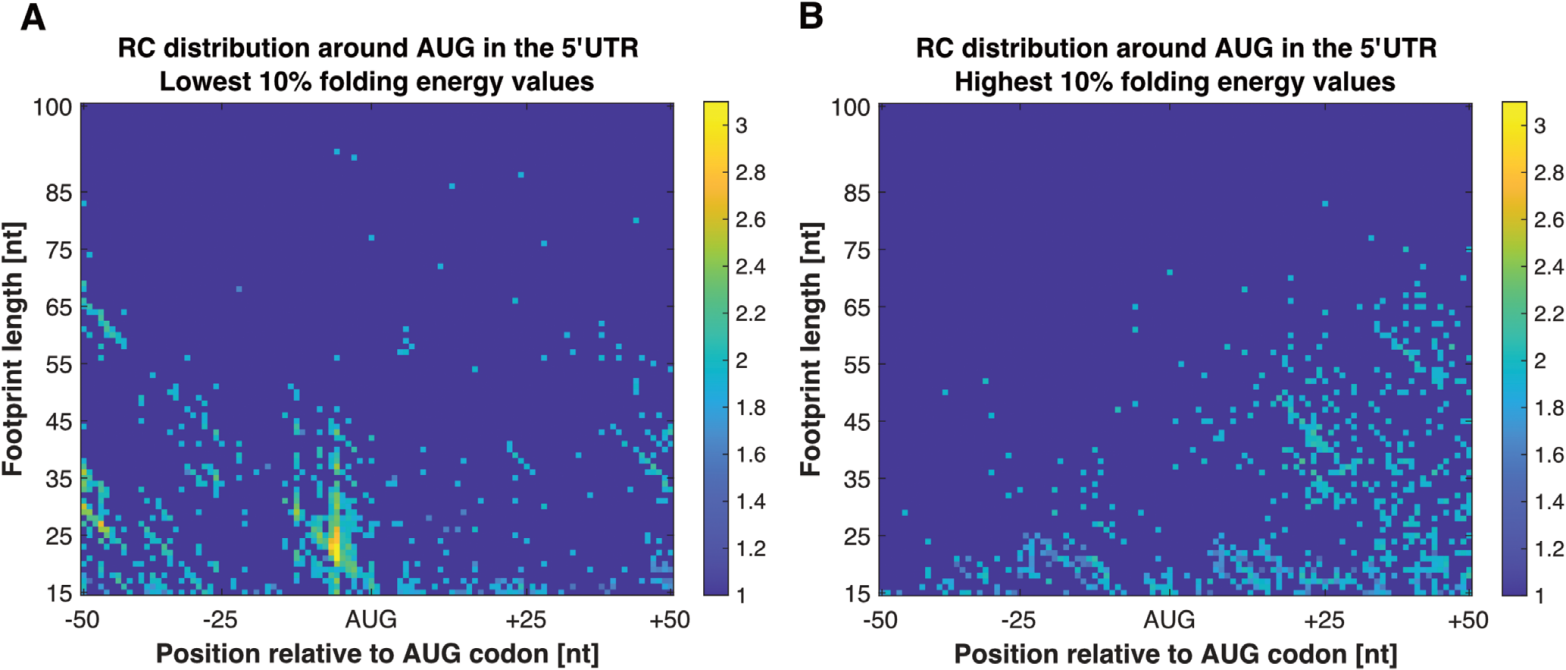
RC distribution surrounding AUGs in the 5′UTR with stable/unstable structure on average in the 20 sliding windows downstream to the AUG. (**A**) RC distribution surrounding uAUGs with stable structure. (**B**) RC distribution surrounding uAUGs with unstable structure.

Aiming to test whether uAUGs located 16–20 nt upstream of stable structure would favor their recognition, we performed the analyses above for uAUGs with high/low folding energy on average in the 20 sliding windows downstream to the AUG. The results show that we tend to see more read counts and higher MIC score (0.3095 compared to 0.1958) in uAUG which are upstream of a stronger mRNA structure (Figure 9). This result suggests that strong folding after a uAUG tends to promote initiation. We also show that the signal is true for long uORF and is weaker for short uORF (Supplementary Figures S12, S13 and Supplementary Tables S6 and S7).

### Motifs in the 5′UTR have an influence on the SSU scan

Many motif-based features were selected for inclusion in predictors that yielded relatively high correlations between predicted RC and real RC (Figure 4). We decided to perform an MIC analysis on those motifs that were most frequently included. The motifs are sequences that were identified using the HOMER (Hypergeometric Optimization of Motif EnRichment) tool (56). HOMER is based on a differential discovery algorithm, as it identifies elements that are specifically enriched in the target set relative to a reference set. We defined all of the 5′UTRs as target set, and created reference set in the following manner: for each 5′UTR we considered a sequence in the same length as the real UTR that is in a gap distance of 100 nucleotides upstream to the beginning of the real 5′UTR. The idea was that since we are trying to find motifs in the 5′UTR, we’ll use reference set that is not UTR, aiming to find motifs that are unique to that region.

For each of the top selected motifs, we calculated its score in sliding windows throughout the 5′UTR (see Materials and methods section). Next, we divided the windows into groups of high and low motif scores, according to the top and bottom 5% values, respectively. Finally, in the same way we constructed the matrix for AUG features in the first section, we constructed matrices for high and low scores of the motif and applied the MIC algorithm to them.

The results show that several motifs have an association with the RC profile distribution around them. The first is related to the poly(A)-binding protein, an RNA-binding protein that triggers the binding of eukaryotic initiation factor 4 complex (eIF4G) directly to the poly(A) tail of mRNA, and is presumed to promote the formation of a closed-loop structure between the mRNA cap and the poly(A) tail (61). Compared to low motif scores, we identified that a high motif score usually produces a higher MIC score (Table 3), and we can see that the RC profile distribution around the motif shows accumulation of RC next to the motif (Supplementary Figure S14). An additional motif that was selected frequently is CG-bias. In this case we see a reverse relation, where high motif score produces lower MIC score and low motif score produces higher MIC score (Table 3).

**Table 3.** MIC analyses of the top selected motifs

Motif score	MIC score	*P* value	Total Number of windows that constructed the signal	Number of points that passes the threshold	Motif logo	Number of times the motif was selected (% of total models)
**Motif: ‘HAAUAAAGNN’**						
High	0.2566	< 0.001	88,328	3442	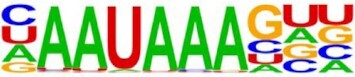	49 (2.85%)
Low	0.1549	< 0.001	88,322	3429		
**Motif: ‘SSSSSSSSSS’**						
High	0.1033	< 0.001	86,888	3604	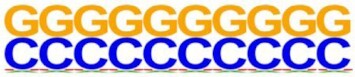	45 (2.62%)
Low	0.2414	< 0.001	88,123	3608		
**Motif: ‘DRUGUUGCAA’**						
High	0.2889	< 0.001	88,322	3553	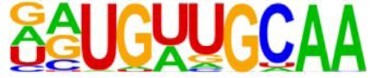	41 (2.38%)
Low	0.1754	< 0.001	88,341	3543		
**Motif: ‘CAUAUAAAAG’**						
High	0.2791	< 0.001	88,324	3702	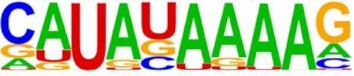	31 (1.80%)
Low	0.1411	< 0.001	88,346	3694		

Comparison of high and low score of motifs selected multiple times. The number of windows that constructed the signal and number of points that passes the threshold were controlled and aligned to all signals. MIC score and *P* value were calculated as described in the Materials and methods section. The results presented in the table are for discretization process that sets the threshold of each row (i.e. footprint length) as the mean (*m*) value of the row.

Interestingly, additional motifs that were selected are those related to transcription factors (TF). First motif was identified as related to C/EBP:AP-1 complex; in *S. cerevisiae*, both C/EBP and AP-1 are sequence specific DNA-binding transcription factors (62). It was previously shown in mammalian cells that this complex activates gene expression through the binding sites TGACGCAA, TGATGCAA and TGTTGCA (as appears in our results) (63). The MIC analysis shows that a higher motif score produces a higher MIC score (Table 3), and from the partial correlation analysis we see mostly positive partial correlation, yet less significant compared to the last motifs (Supplementary Figure S15). The second was the motif of TATA-box, which is a binding site to several transcription factor (64). The MIC analysis shows that a higher motif score resulted in a higher MIC score (Table 3). However, the partial correlation between the motif and the RC is not unequivocal (Supplementary Figure S15).

## DISCUSSION

In this study, based on direct measurements of the SSU footprints from TCP-seq data, we identified several transcript features that affect the SSU movement along the 5′UTR, and created for the first time a computational predictive model of the SSU density. We are the first to model this type of data in order to better understand translation initiation in *S. cerevisiae* and provide a novel approach for its analysis based on MIC.

The prediction of the SSU density based on transcript features yielded relatively low yet significant correlations. These results meet our expectations since the correlation between two vectors, each hundreds of thousands of values long, is not expected to be high, especially when the data at such resolution is sensitive to noises and biases (45). In addition, since it is a complex model and there are probably dozens of variables that affect the SSU scan, we believe that it is not trivial to get such significant correlations as we did, even if those obtained are relatively low.

We analyzed here all footprint length ranges from 17 to 100 nt, as was done in previous studies based on TCP-seq (26). Shorter footprint lengths (∼15–17 nt) are probably a result of strong digestion of longer footprint lengths. Nonetheless, as we mentioned earlier, it was suggested that 19 nucleotides are probably already a specific configuration of the SSU conceivably derive from paused SSU still in open, scanning-competent configuration.

In addition, note that the predictions for the different footprint lengths are independent (the prediction was performed for each FP length separately). Thus, the results in Figure 3B,C are presented on the same graph such that it will be able to see the entire image and try to conclude for different footprint lengths.

Examining the distribution of the features that were selected in various footprint lengths showed that different footprint lengths produce different feature selection distributions. Thus, this may suggest that different conformations of the SSU and the initiation factors tend to have different interaction efficiencies with features of the UTR.

Furthermore, several features that affect the SSU RC were identified to be robust to the footprint lengths; for example, in case of the 5′UTR length, the partial correlation between the feature and the SSU density resulted in inverse relation in many footprint lengths, meaning shorter UTRs tend to have higher RC. A possible explanation for this result may be that the length of the 5′UTR is shorter in highly expressed genes (65,66); in addition, highly expressed genes undergo selection for high initiation rates (5,14,27,67–69), which may be expressed as high SSU density in the 5′UTR. An additional explanation is the fact that the probability of abortion of the SSU increases as the distance from the 5′ of the UTR increases, promoting higher densities for shorter UTRs (similarly to complete ribosomes (23)). The aforesaid explanation is also relevant to the 5′UTR length relative to the ORF length, which was also selected as a feature in several predictors; this result may be partially related to the fact that highly expressed genes tend to undergo selection to be more compact, in order to minimize cellular resources (70,71). Previous study (43) also found the 5′UTR length as relevant feature. Note however that the definitions and analysis used here and in (43) are different: for example, in our analysis we control for dozens of additional features and analyse the RC in each window in the UTR while in (43) there is no such control and the RC is compared to the RC surrounding the main AUG.

Additional features that were selected numerous times are features related to uAUG, including a binary feature that describes whether there is or isn’t an AUG in the current sliding window, and the average distance of uAUGs in the current sliding window to the main AUG START codon. The analysis of the binary feature showed that if there is an AUG in the sliding window, there are more RC in the window. The analysis of the average distance to the main AUG START codon feature yielded positive partial correlations between the feature and the real RC, meaning that for longer distance to the main AUG (i.e. more upstream) there are more RC. This may be because there is a selection for AUGs with low AUG context score in the vicinity of the main AUG START codon (60) that has an impact on the number of RC. In addition, as was mentioned above, this may be due to the assumption that the probability of abortion of the SSU increase as the distance from the 5′ end of the UTR increases.

An interesting hypothesis that arises from the features analyses is that when the SSU reaches a strongly folded region, it detaches from the mRNA, probably due to its limited ability to disrupt the strongly folded secondary structure (38,39). In conclusion, we believe that these features have an effect on the SSU scan and translation initiation and are an indication that the efficiency of translation initiation is encoded in the transcript.

As was mentioned above, the SSU RC coalesced into three major sizes (19, 29 and 37 nt) at the main AUG start codons. Examining these specific FP lengths, we identified that the correlations between the predicted RC (based on our model) and the real RC is relatively high for all these three lengths: 0.1868 for 19 nt, 0.1715 for 29 nt and 0.1672 for 37 nt. We found similarities but also some differences between the features and signals related to these three clusters of FP lengths. In all three cases, for example, features that are related to the appearances of the AUG triplet, the 5′UTR length, and local mRNA folding had high ranking.

However, we also found some aspects that are unique to each of these footprint lengths such as: the score of the motif ‘AAAAAGGC’ for length 19 nt, the score of the motif ‘GGCGGCUG’ for length 29 nt and the score of the motif ‘KGMCAGCUND’ for 37 nt (Supplementary Table S2). Thus, we conclude that similar but not identical models are needed for predicting the RC density of each of the major SSU conformations.

We showed that AUGs in the 5′UTR with high context scores induce an RC distribution similar to the RC surrounding the main AUG, therefore postponing the SSU scan. A previous study found that strong uAUG context is associated with stronger translational inhibition (41). Nevertheless, we are the first to provide direct large-scale evidence that uAUG context score in endogenous genes and in natural conditions has an influence on the SSU scan. Our analysis may suggest that AUG context affects the small sub-unit movement and contribute to ∼50% of the variance related to the RC distribution near the main start codon and over 17% near UTR AUGs with high context score. Other codes surrounding the main AUG start codon include additional sequence and structural features, many are reported in this paper, for example: the local mRNA folding energy, the 5′UTR length, additional uAUG codon, etc. In addition, we show that longer uORFs in the 5′UTR also induce an RC distribution that is more similar to the RC surrounding the main AUG compared to short uORFs, probably due to additional/stronger signals surrounding the AUG that are related to triggering translation. Our analysis suggest that the TCP-IC measurement can be generalized in order to predict if uORFs are functional.

We also show that there are more RC and higher MIC in uAUG which are upstream of a stronger mRNA structure. This result suggests that strong folding downstream to an AUG codon in the 5′UTR may improve codon recognition. Nevertheless, it is also possible that once the SSU reaches an uAUG with strong folding downstream, it may detach from the mRNA, as we suggested earlier.

Several motifs were selected by the predictors that yielded relatively high correlations between the predicted RC and the real RC. Therefore, we have applied the MIC analysis on top selected motifs, comparing between high and low motif scores. The results show that some motifs have an influence on the SSU RC profile distribution; first is related to the poly(A)-binding protein (PABP), which is an RNA-binding protein. While PABP is known to bind to the 3′UTR and affect polyadenylation process, studies in the field have shown that PABP can also bind to the 5′UTRs (72,73). Since the motif AAUAAA is similar to a polyA tail it is natural and probable that the PABP will bind to it (even if it is in at the 5′UTR).

However, this does not mean that a PABP motif that appears in the 5′UTR will not attract the PABP that will affect the SSU movement. From the MIC analyses and the visual differences between the high and low motif scores signals, we can see that for higher motif scores there is an accumulation of RC next to the motif. Although the protein is typically bound to the 3′UTR, previous studies in the field have shown that it can bind to sequences in the 5′UTR (72,73), thus resulting in an increase in the number of the SSU RC. The partial correlations of the motif with the RC are positive, supporting the hypothesis that higher motif score results in more RC. Note that it is possible that this motif directly affects the SSU, but it is also possible that the affect is indirect: the motif causes the binding of the poly(A)-binding protein which increases the density of the SSU e.g. via interacting with it and/or due to delaying it. In addition, it is possible that the motifs that affect the SSU are similar to the PABP motif but that there are no direct interactions between the PABP and the SSU.

An additional motif that was selected many times is CG-bias. In this case, we observed a reverse relationship, where a high motif score produces a lower MIC score. These results meet our expectations, as strong CG bias may be indicative for strong mRNA folding that, as we suggested, causes the SSU to detach from the mRNA.

Interestingly, additional motifs that were selected are motifs related to TF binding sites. The MIC analyses of these motifs show that higher motifs scores result in higher MIC scores, meaning the SSU lingers at these binding sites. There are a number of possible explanations for the effect of binding sites of transcription factors on the SSU scan: first, RNA is similar to DNA in several aspects. It is possible that transcription factors attach to the mRNA, probably with weaker affinity, yet one that is enough to ‘disturb’ the SSU scan and increase its density. Second, it is possible that the TF and the SSU proteins are biochemically similar. This would be significant even if only parts of their domains have resemblance. It is possible that they are similar due to co-evolution, which would present coupling between transcription and translation processes. In that case, a binding site for transcription factor also will have an influence on translation. Note that it is also possible that they are simply similar in terms of structure and not function.

The results from the last section are interesting in themselves, but also demonstrate the power of the tools we have proposed in this study to understand the effect of different features on the scanning of the small subunit.

In conclusion, using TCP-seq data and computational analyses, we have presented in this study new insights on translation initiation in *S. cerevisiae* and possibly other eukaryotes while providing quantitative measures to our claims. Our results have an implication on different research fields. We discovered features that affect the SSU scan and as a result affect translation initiation in *S. cerevisiae*, allowing better understanding of this process. Our computational approach was tailored to deal with the fact that the TCP-seq data are noisy and various variables can be related to the SSU movement. However, further extensive experimental validation should be performed to fully understand the reported discoveries. The most relevant validations should include TCP-seq experiments on various engineered reporter proteins which include various versions of the discovered signals in their UTRs.

We believe that our findings can be implemented to engineered transcripts for efficient translation initiation. In addition, this study encourages the performance of the same novel quantitative analyses on additional different organisms in order to gain insight into the complex process of translation.

The results presented in this study suggest further interesting research directions. First, the TCP-seq experiment was performed on the model organism *S. cerevisiae*. It will be interesting to generalize the results to other eukaryotes organisms when such data are available, and furthermore, to study the differences between the different organisms, between different cell cycle phases (e.g. as was done in (74,75)), and how it affects cancer mutations. Second, it will be interesting to generate a library of heterologous genes, insert the features we identified as important and perform additional TCP-seq, in order to check the causality (e.g. as was done in (32,33) for studying protein levels). Third, our study is a proof of concept that TCP-seq data can be used in order to predict the SSU density. Our model is based on a basic linear regression, while using a naïve feature selection process, in order to keep it simple while trying for the first time to use that kind of complex data set. Further future research should use more complex machine learning algorithms to achieve better results.

For example, our analyses can be implemented as a follow-up study to (76), where the authors carried out a detailed study on how synthetic 5′-UTRs can alter gene expression in *S. cerevisiae*. They focused on a single gene (CYC1) and built 58 synthetic variants via single and multiple point mutations between positions −15 and −1, then quantified the strength of the synthetic leader sequences using fluorescence measurements.

They showed that the leader configuration upstream of the Kozak sequence has a marked influence on translation initiation and one central mechanism that they suggest is via the effect of the mutations on local mRNA folding near the AUG codon which fits our conclusions regarding the effect of local folding on SSU RC. We believe that such an experiment combined with TCP-seq for some of the variants and with the analysis suggested here is a useful tool for studying causal relations related to translation initiation.

## Supplementary Material

gkac021_Supplemental_File
