# Supplementary material for: Modeling the ribosomal small subunit dynamic in Saccharomyces cerevisiae based on TCP-seq data
Source: Nucleic Acids Res. 2022 Jan 31;50(3):1297–316. doi: 10.1093/nar/gkac021 (PMC8860609; doi:10.1093/nar/gkac021)
Supplement: gkac021_Supplemental_File [file gkac021_supplemental_file.pdf]

Modeling the ribosomal small subunit dynamic in *S. cerevisiae* based on TCP-seq data

Supplementary Information

Tamar Neumann [1], Tamir Tuller [1,2*]

[1] Department of Biomedical Engineering, Tel Aviv University, Tel Aviv 6997801, Israel.

[2] The Sagol School of Neuroscience, Tel-Aviv University 6997801, Tel Aviv, Israel.

* E-mail: tamirtul@post.tau.ac.il (TT)

Supplementary methods

Features:

GC content

GC content is the percentage of nitrogenous bases on a DNA or RNA molecule that are either guanine or cytosine. GC pairs are bound by three hydrogen bonds, while AT pairs are bound by two hydrogen bonds. Thus, DNA with high GC-content is more stable than DNA with low GC-content. This feature represents the GC content in the current sliding window.

Nucleotide distribution

The frequency of each nucleotide in the sliding window (4 features).

Groups of two nucleotides distribution

The frequency of each nucleotide pair in the sliding window (16 features).

Groups of three nucleotides distribution

The frequency of each group of three nucleotides in the sliding window; not referred as codons since they appear in 5’UTR (64 features).

Supplementary figures:

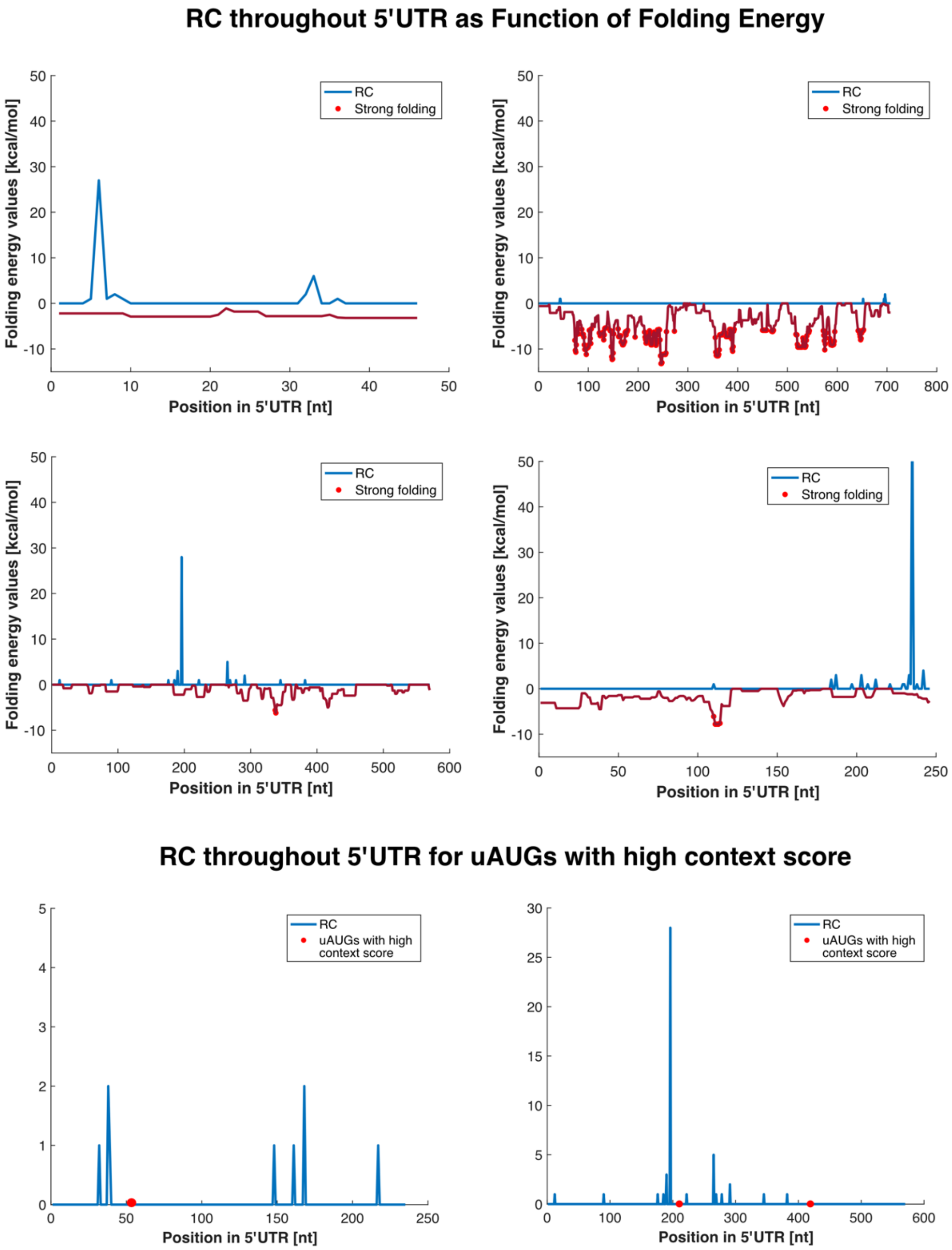

Figure S1. *Examples for sequences with RC throughout the 5’UTR as function of two features: folding energy and uAUG with high context score.*

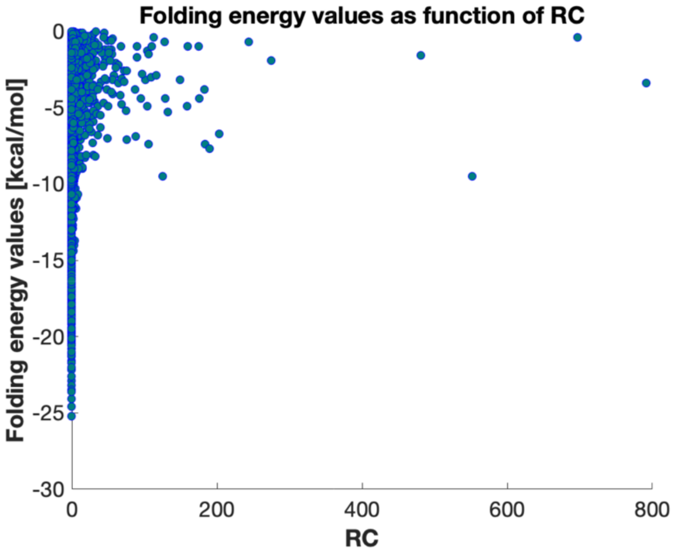

Figure S2. Folding energy values as calculated by MATLAB rnafold function vs RC . *Folding energy values are presented in units of [kcal/mol]*

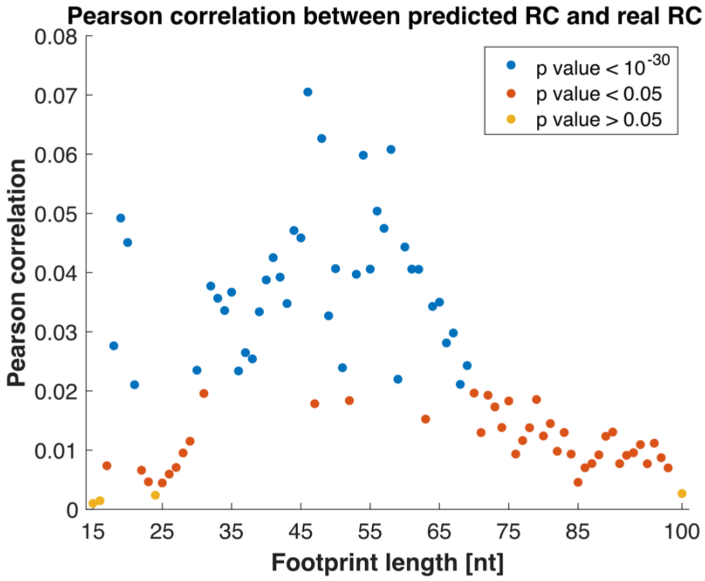

Figure S3. Prediction of the SSU density based on transcript features. *Pearson correlation between the predicted RC and the real RC, as function of footprint length. The presented values are the median correlations obtained from all 20 predictors in each footprint length, and the colors corresponds to the p values. The correlations are lower compared to Spearman correlations, as Pearson correlation is strongly biased towards linear trends. Yet, the results yielded significant p values, an indication for stability.*

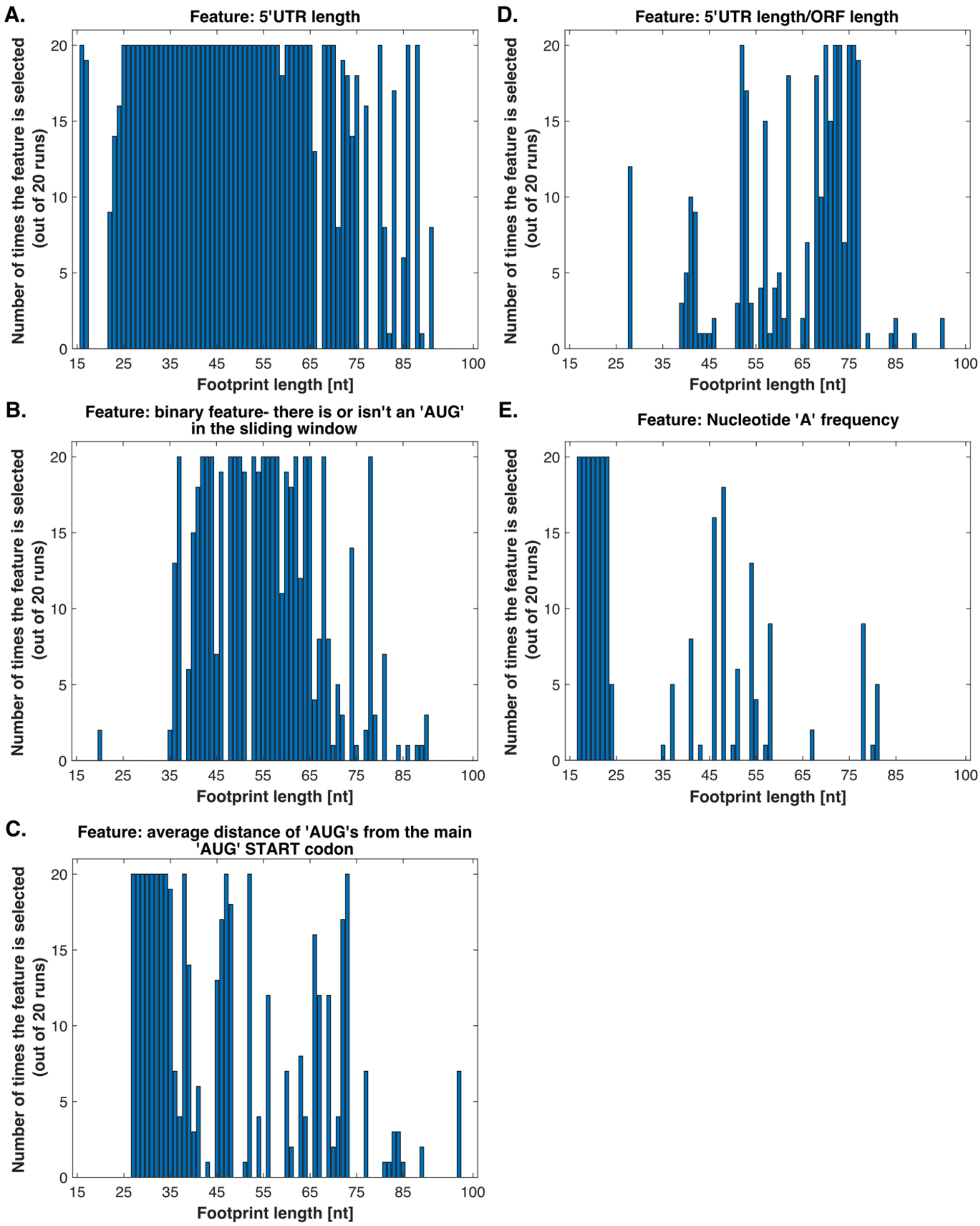

Figure S4. Features selected by multiple predictors as function of footprint length. *Number of times each feature was selected (out of 20 predictors) in all footprint lengths.*

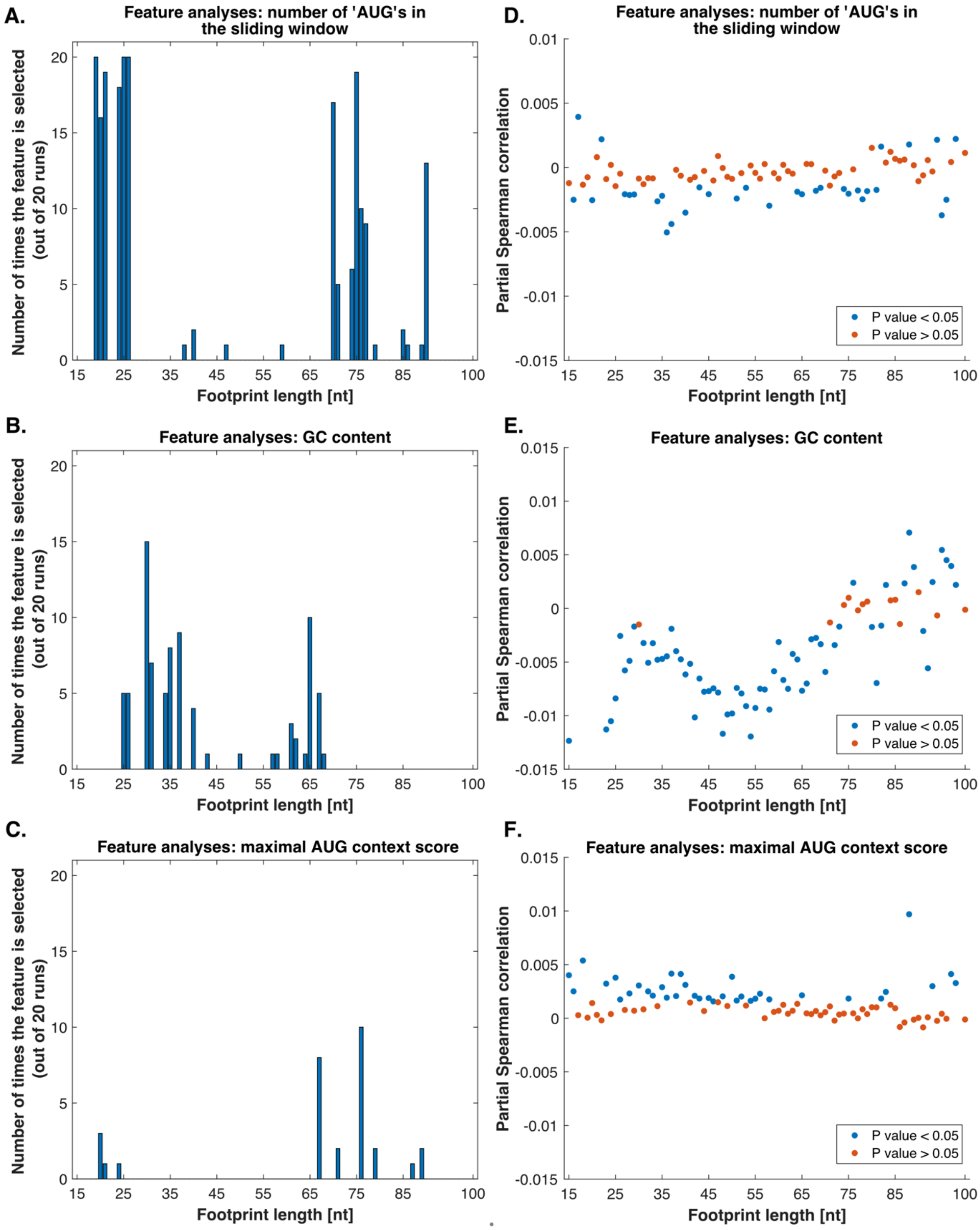

Figure S5. Features selected by multiple predictors as function of footprint length and the partial correlation of each feature with the SSU read counts. A-C. *Number of times each feature was selected (out of 20 predictors) in all footprint lengths.* ***D-F.*** *Partial Spearman correlation between each feature and the SSU RC, while controlling for the other features and the mRNA levels.*

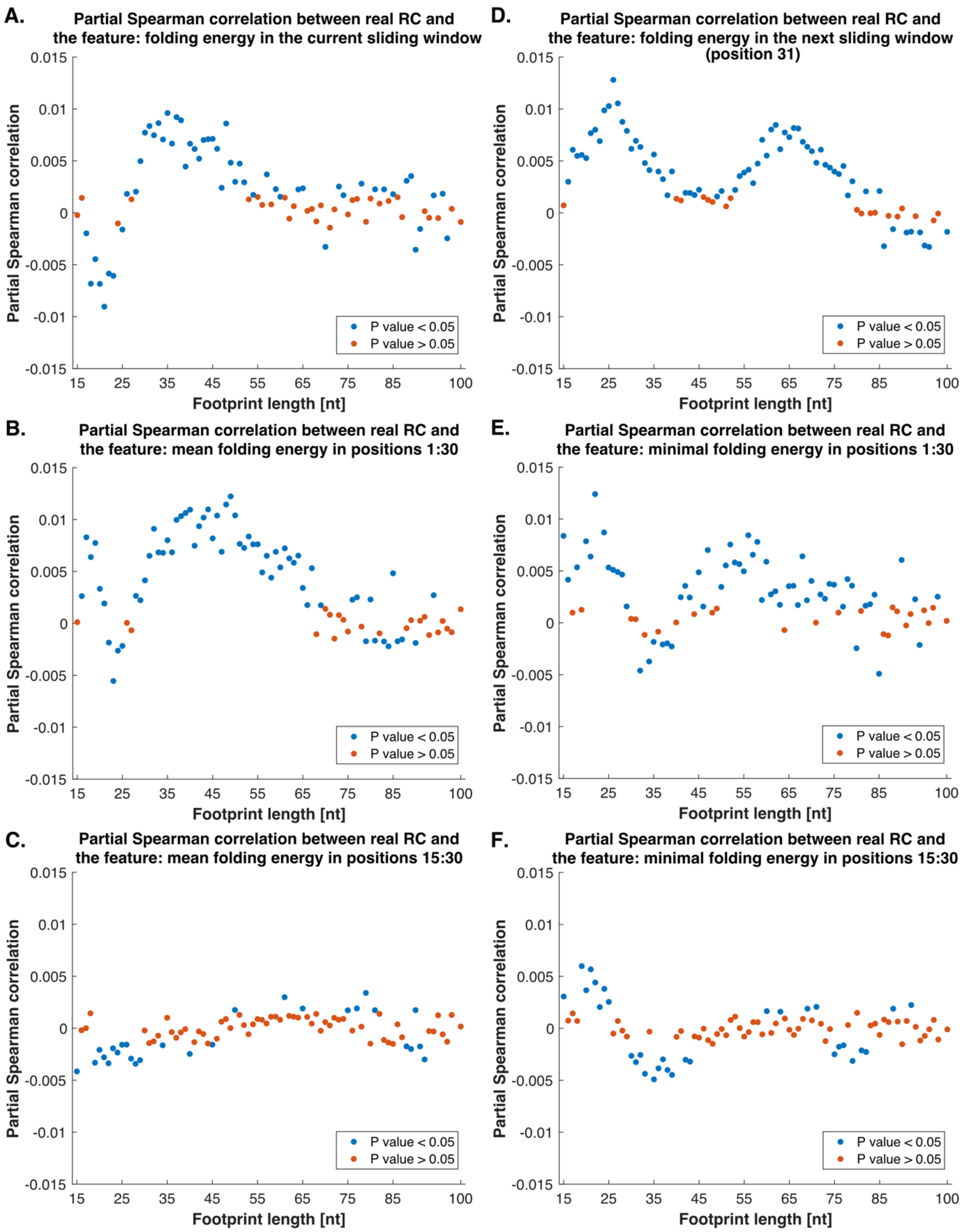

Figure S6. Partial Spearman correlation between folding energy features and the real RC, while controlling for the other features and mRNA levels, as function of footprint length.

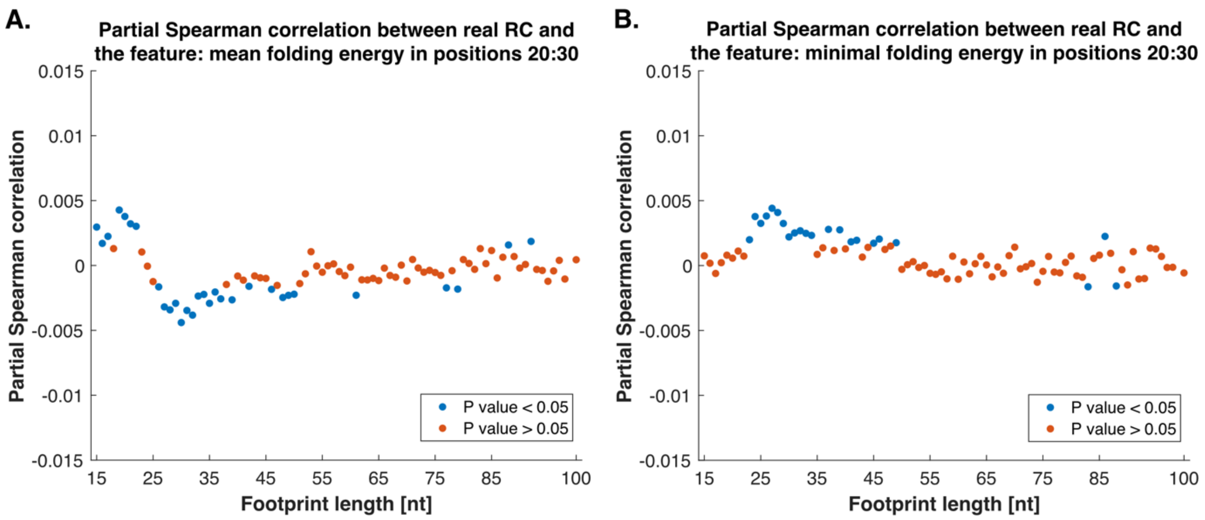

Figure S7. Partial Spearman correlation between folding energy features and the real RC, while controlling for the other features and mRNA levels, as function of footprint length*.*

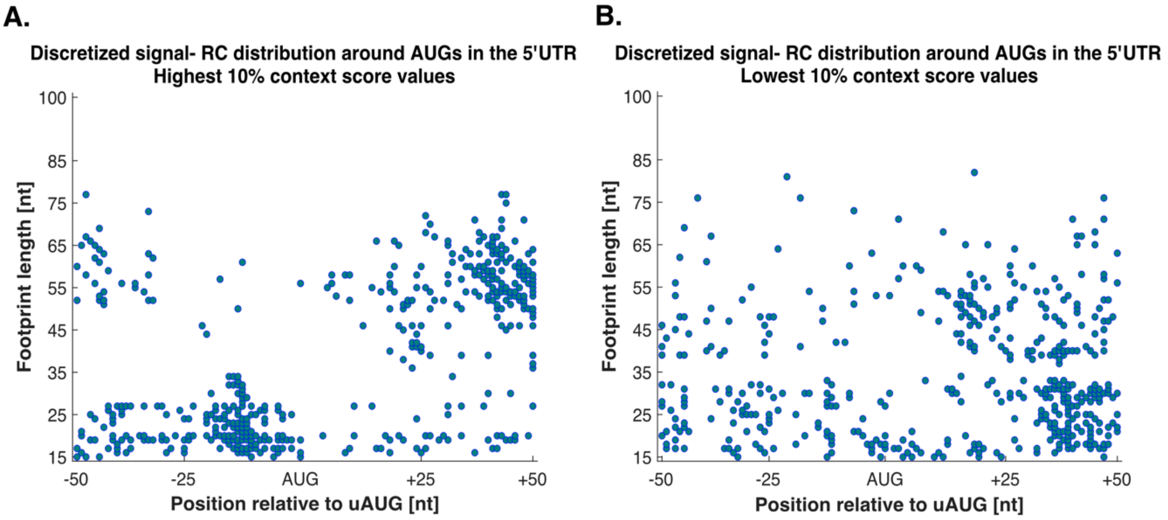

Figure S8. RC distribution surrounding AUGs in the 5'UTR with high/ low AUG context score, after discretization process. *Using discretization process that sets the threshold of each row (i.e. footprint length) as the threshold that optimizes the MIC score of the entire matrix. The discretization process transfers the matrix from a grey scale image to a binary one, a process that enables us to apply computational tools such as MIC on the data.*

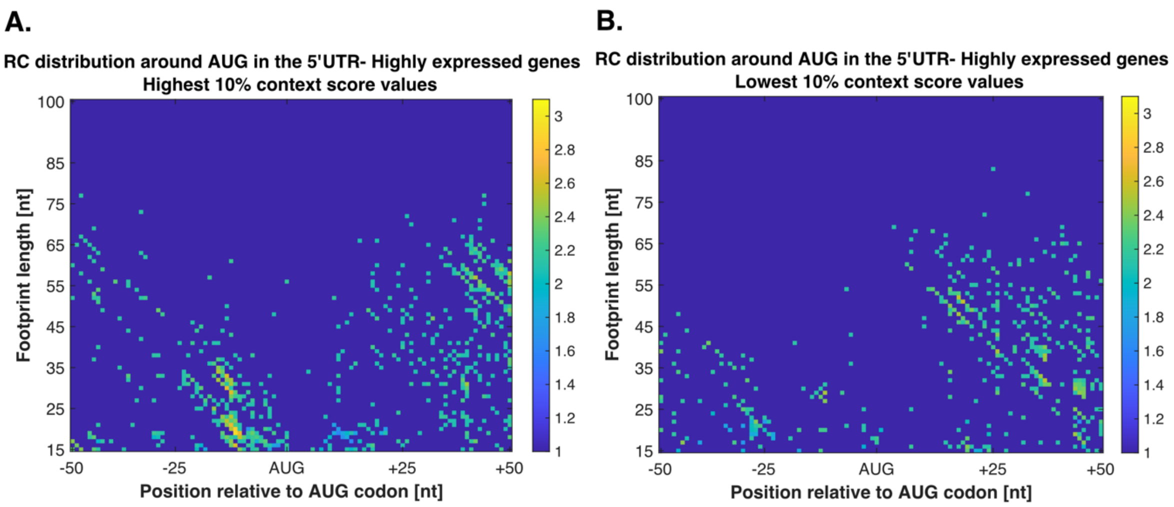

Figure S9. RC distribution surrounding AUGs in the 5'UTR with high/ low AUG context score in highly expressed genes. A. *RC distribution around AUGs in the 5'UTR with high AUG context score. It can be seen that AUGs with high context score present a trend that is more similar to the RC distribution surrounding the main AUG start codon.* ***B.*** *RC distribution around AUGs in the 5'UTR with low AUG context score. No trend was identified, neither by the scatterplot or by the MIC score analyses. The results stay robust to different selections of genes.*

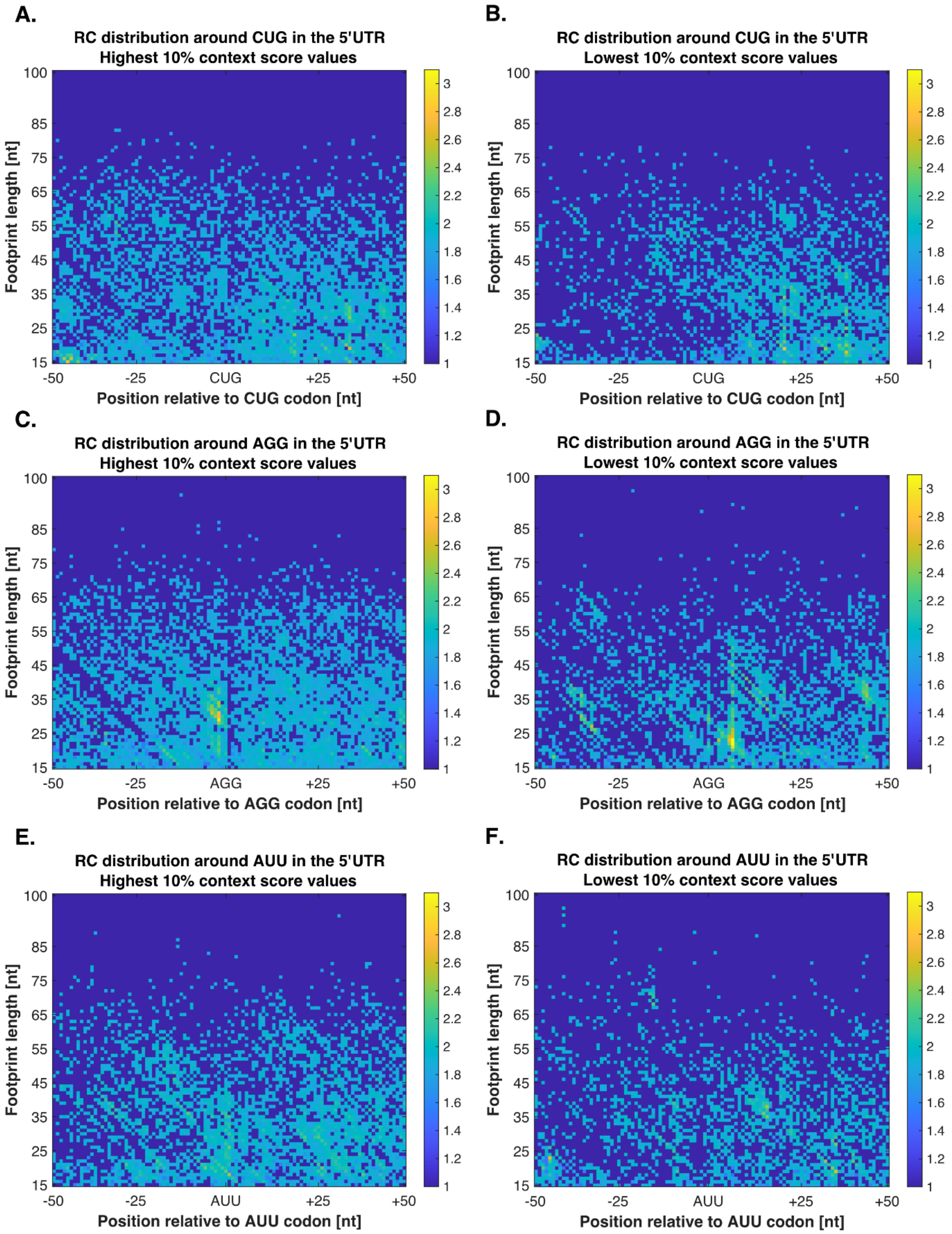

Figure S10. RC distribution surrounding AUG-like codons in the 5'UTR with high/ low AUG context score. A-B. *RC distribution around CUGs in the 5'UTR with high/low AUG context score.* ***C-D.*** *RC distribution around AGGs in the 5'UTR with high/low AUG context score.* ***E-F.*** *RC distribution around AUUs in the 5'UTR with high/low AUG context score.*

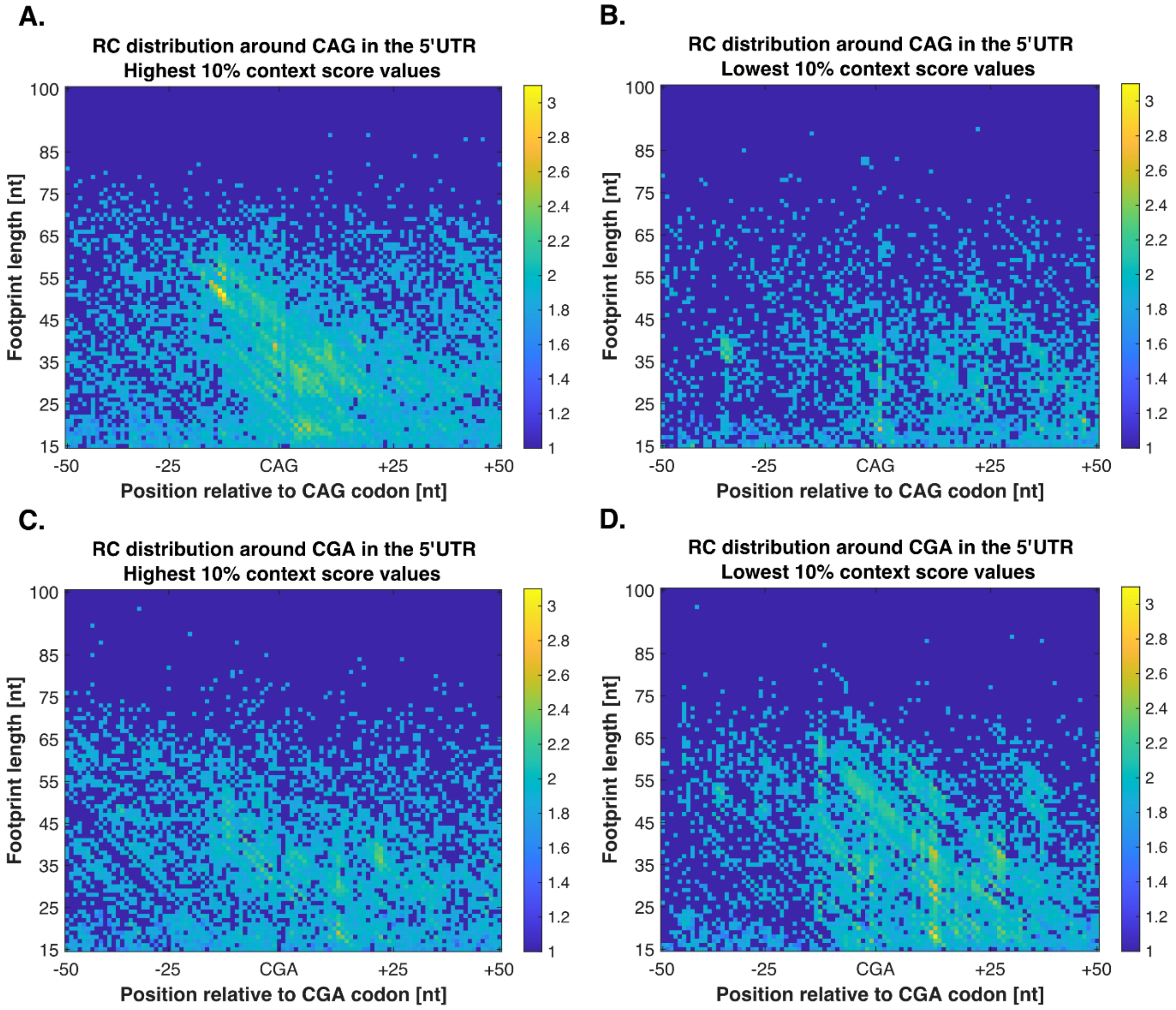

Figure S11. RC distribution surrounding additional codons in the 5'UTR with high/ low AUG context score. A-B. *RC distribution around CAGs in the 5'UTR with high/low AUG context score.* ***C-D.*** *RC distribution around CGAs in the 5'UTR with high/low AUG context score.*

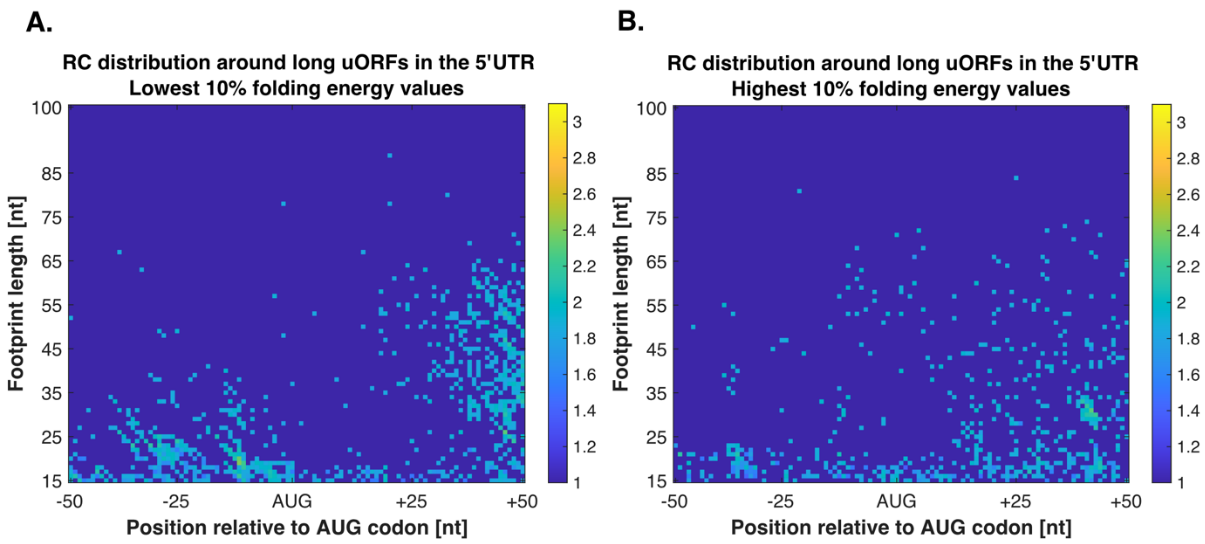

Figure S12. RC distribution surrounding AUGs in the 5'UTR with long distance to the nearest stop codon, comparison between with stable/unstable structure on average in the 20 sliding windows downstream to the AUG. A. *RC distribution surrounding uAUGs with stable structure.* ***B.*** *RC distribution surrounding uAUGs with unstable structure.*

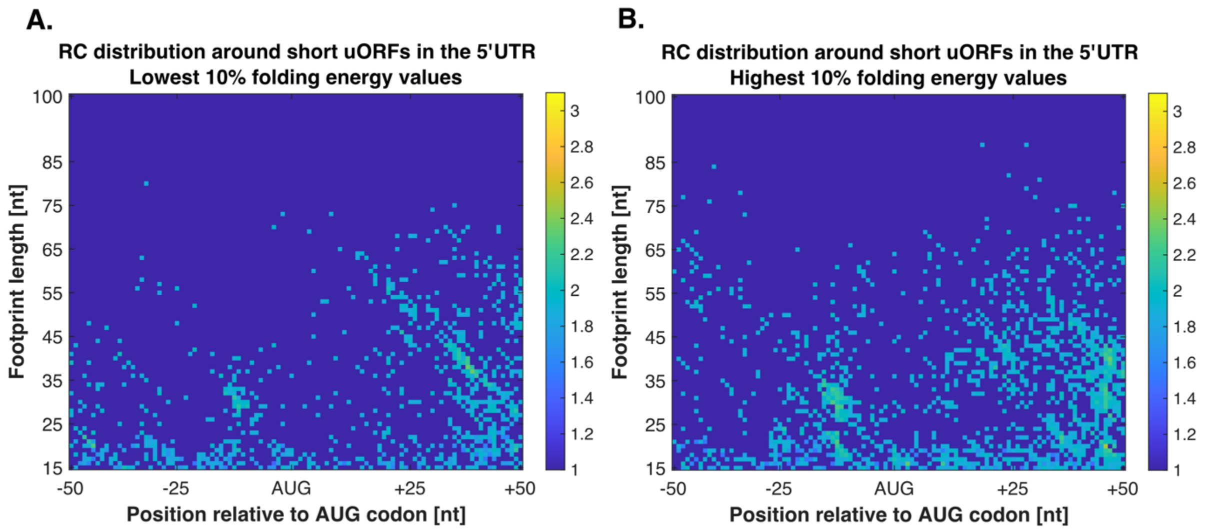

Figure S13. RC distribution surrounding AUGs in the 5'UTR with short distance to the nearest stop codon, comparison between with stable/unstable structure on average in the 20 sliding windows downstream to the AUG. A. *RC distribution surrounding uAUGs with stable structure.* ***B.*** *RC distribution surrounding uAUGs with unstable structure.*

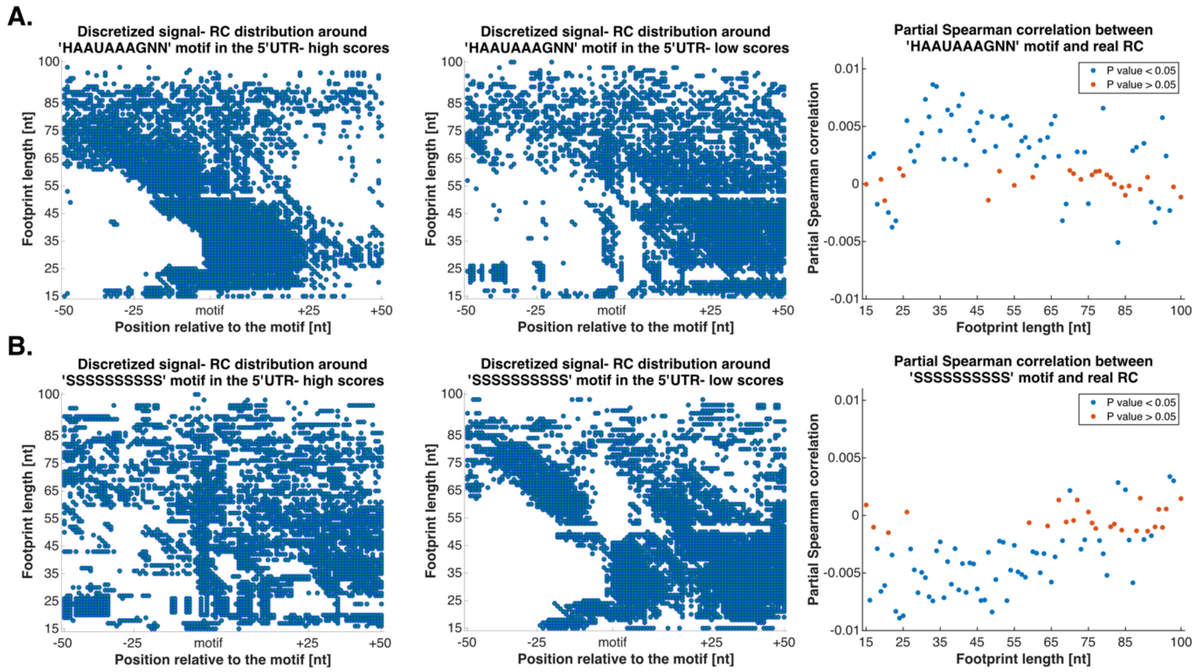

Figure S14. RC distribution surrounding motifs in the 5'UTR with high/ low motif score. A. *RC distribution around the motif 'HAAUAAAGNN' with high and low scores, and the partial correlation of the motif with the SSU RC, controlling for the other features and mRNA levels. It can be seen that for windows with high motif score there is an accumulation of RC next to the motif, in contrast to windows with low motif score.* ***B.*** *RC distribution around the motif 'SSSSSSSSSS' with high and low scores, and the partial correlation of the motif with the SSU RC, controlling for the other features and mRNA levels. For the second presented motif we see reverse relation from the first one, as for windows with high motif score no trend was identified, compared to the low score case. For all figures, the presented results are for discretization process that sets the threshold of each row (i.e. footprint length) as the mean (m) value of the row.*

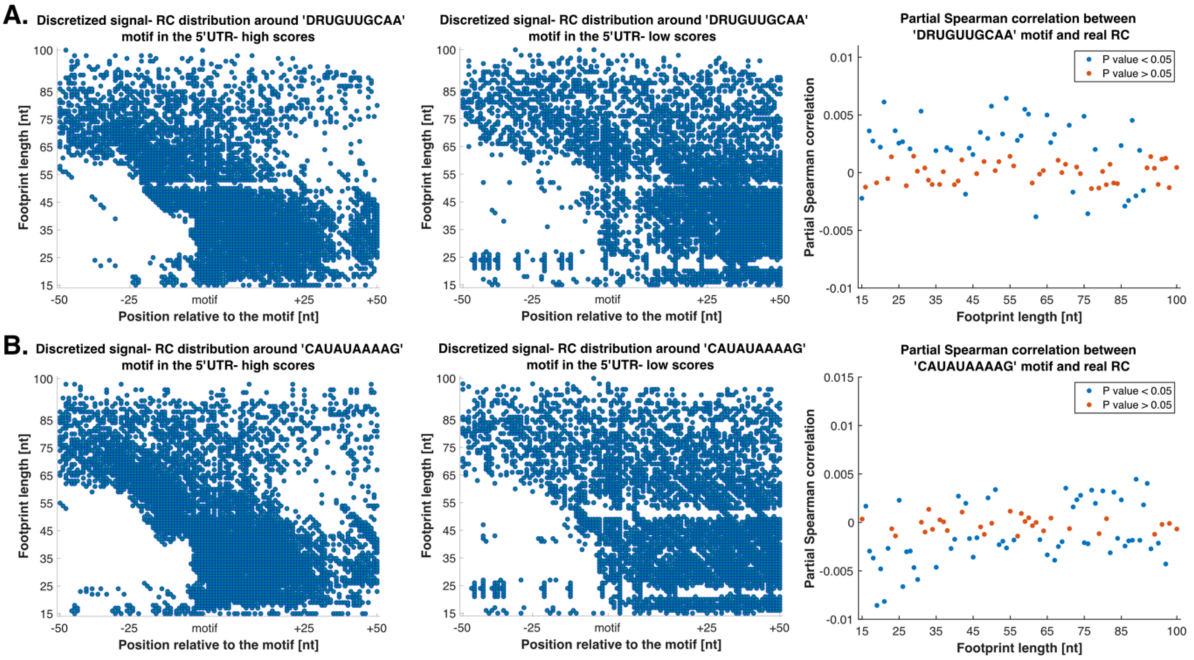

Figure S15. RC distribution surrounding motifs in the 5'UTR with high/ low motif score, and the partial correlation of the motif with the SSU RC, controlling for the other features and mRNA levels.

Supplementary tables:

Feature	Total number of appearances	% Out of total predictors
5'UTR length	1163	67.62
Binary feature that describes whether there is or isn't an AUG in the current sliding window	583	33.90
The average distance to the main AUG START codon	468	27.21
5'UTR length/ORF length	321	18.66
Mean folding energy in the current sliding window (positions 1:30)	249	14.48
Nucleotide 'A' frequency	245	14.24
Number of 'AUG's in the current sliding window	202	11.74
Folding energy in the current sliding window (position 1)	106	6.16
Minimal folding energy in the current sliding window (positions 1:30)	96	5.58
Number of 'AG's in the current sliding window	91	5.29
GC content	84	4.88
Average AUG context score in the current sliding window	84	4.88
Nucleotide 'C' frequency	65	3.78
Mean folding energy in positions 15:30	62	3.60
Number of 'AAA's in the current sliding window	56	3.26
The maximal score of the motif 'HAAUAAAGNN'	49	2.85
Number of 'UUU's in the current sliding window	48	2.79
The maximal score of the motif 'SSSSSSSSSS''	45	2.62
Number of 'CCU's in the current sliding window	42	2.44
The maximal score of the motif 'DRUGUUGCAA'	41	2.38
Number of 'CUG's in the current sliding window	40	2.33
Number of 'UG's in the current sliding window	33	1.92
The maximal score of the motif 'CACCGCUU'	33	1.92
The maximal score of the motif 'AUUUGCAUAACAAUG'	32	1.86
The maximal score of the motif 'CAUAUAAAAG'	31	1.80
Maximal AUG context score in the current sliding window	30	1.74
Number of 'CC's in the current sliding window	29	1.69
The maximal score of the motif 'CCCCGCGC'	29	1.69
Number of 'AAG's in the current sliding window	27	1.57
Number of 'AA's in the current sliding window	27	1.57
Mean folding energy in positions 20:30	27	1.57
The maximal score of the motif 'CCAGGAACAG'	25	1.45
Number of 'CU's in the current sliding window	24	1.40
Minimal folding energy in positions 20:30	24	1.40
Number of 'UCC's in the current sliding window	23	1.34
The maximal score of the motif 'KGMCAGCUND'	23	1.34
Number of 'CAA's in the current sliding window	22	1.28

Number of 'GGG's in the current sliding window	22	1.28
The maximal score of the motif ‘AAAAAGGC’	22	1.28
The maximal score of the motif ‘GUGGDCCYNNNNNNN’	22	1.28
Minimal folding energy in positions 15:30	21	1.22
Number of 'AGC's in the current sliding window	20	1.16
Number of 'CUC's in the current sliding window	20	1.16
Number of 'UAC's in the current sliding window	20	1.16
Number of 'CAU' in the current sliding window	19	1.10
Number of 'UGU' in the current sliding window	19	1.10
The maximal score of the motif ‘AUAUAGUAGAAG’	19	1.10
Number of 'CAG' in the current sliding window	18	1.05
Number of 'CGC' in the current sliding window	18	1.05
Number of 'UGC' in the current sliding window	18	1.05
AUG context score divided by the context score of the main AUG START codon	18	1.05

Table S1. Ranking of the features according to their total number of appearances*. The table summarizes the information about the top selected features, the total number of times each feature was selected, both in number and percentage relative to the total number of predictors (20 predictors in each footprint length, 86 footprint lengths, total number of 1720 predictors).*

	**Correlation**	**Top rated feature**	**Second rated feature**	**Third rated feature**	**Fourth rated feature**
**19 nt**	0.1868	Number of times an ‘AUG’ appears	Nucleotide ‘A’ frequency	Number of times a ‘UUU’ appears	maximal score of the motif ‘AAAAAGGC’
**29 nt**	0.1715	5’UTR length	The average distance of ‘AUG’s in the sliding window from the main AUG start codon	the maximal score of the motif ‘HAAUAAAGNN’	the maximal score of the motif ‘GGCGGCUG’
**37 nt**	0.1672	5’UTR length	Binary feature that describes whether there is or not an ‘AUG’ in the current sliding window	the maximal score of the motif ‘KGMCAGCUND’	GC content.

Table S2. Reported signals in 3 main FP length (19, 29 and 37 nt). *Correlation between real RC (TCP-seq data) and predicted RC. Similar but not identical models are needed for predicting the RC density of each of the major SSU conformations.*

	Optimization	Mean	2*Mean	Median	Mean + std
**Main AUG codon**	0.8795	0.4689	0.5733	0.3567	0.6937
**AUG's with High AUG Context score**	0.5230	0.1784	0.2117	0.1410	0.2683
**AUG's with Low AUG Context score**	0.1389*	0.1092	0.1417	0.1004	0.2060

Table S3. AUGs in the 5'UTR with higher context score has a higher MIC score, similar to the MIC score of the main AUG start codon*. The number of AUGs that constructed the signal (see Materials and methods section) and number of points that passes the threshold were controlled and aligned to all signals. Number of points passes the threshold refers to a control process we performed, aiming to show that the obtained MIC score is not a result of different number of points remaining after the discretization process was completed. Further details can be found in the Materials and methods section. MIC score and p value were calculated as described in the Methods section. The results presented in the table are for AUGs that don't have additional AUG 50 nucleotides upstream and downstream. *p value > 0.05.*

	MIC score for high context score	MIC score for low context score
**AUG**	0.5230	0.1389*
**CUG**	0.3188	0.1724
**AUU**	0.1594	0.1683
**AGG**	0.1207	0.1605

Table S4. Comparison between AUG and AUG-like codons with high/low AUG context score. ** p value > 0.05. Using the MIC algorithm with the optimization process (during the discretization process we set the threshold of each row as the threshold that optimizes the MIC score of the entire matrix). We controlled the number of points that passes the threshold to be aligned with the previous results that we had, so the new results will not be affected by the number of points passes the threshold.*

	MIC score for short uORFs	MIC score for long uORFs
**AUG**	0.1990	0.2838
**CUG**	0.1792	0.2991
**AUU**	0.1200	0.4220
**AGG**	0.1407	0.1339

Table S5. Comparison between AUG and AUG-like codons with short/long distance to the nearest stop codon. *Using the MIC algorithm with the optimization process (during the discretization process we set the threshold of each row as the threshold that optimizes the MIC score of the entire matrix). We controlled the number of points that passes the threshold to be aligned with the previous results that we had, so the new results will not be affected by the number of points passes the threshold.*

	Optimization	Mean	2*Mean	Median	Mean + std
**With stable structure**	0.7196	0.3675	0.4086	0.3418	0.3988
**With unstable structure**	0.1617*	0.1314*	0.1298*	0.1646	0.1669*

Table S6. MIC score resulted for RC distribution surrounding AUGs in the 5'UTR with long distance to the nearest stop codon, comparison between with stable/unstable structure on average in the 20 sliding windows downstream to the AUG. *The number of points that passes the threshold were controlled and aligned to all signals. Number of points passes the threshold refers to a control process we performed, aiming to show that the obtained MIC score is not a result of different number of points remaining after the discretization process was completed. Further details can be found in the Materials and methods section. MIC score and p value were calculated as described in the Methods section. The results presented in the table are for AUGs that don't have additional AUG 50 nucleotides upstream and downstream. *p value > 0.05.*

	Optimization	Mean	2*Mean	Median	Mean + std
**With stable structure**	0.5216	0.1522	0.1575	0.1509	0.2639
**With unstable structure**	0.2332	0.1680	0.2383	0.1225	0.2562

Table S7. MIC score resulted for RC distribution surrounding AUGs in the 5'UTR with short distance to the nearest stop codon, comparison between with stable/unstable structure on average in the 20 sliding windows downstream to the AUG. *The number of points that passes the threshold were controlled and aligned to all signals. Number of points passes the threshold refers to a control process we performed, aiming to show that the obtained MIC score is not a result of different number of points remaining after the discretization process was completed. Further details can be found in the Materials and methods section. MIC score and p value were calculated as described in the Methods section. The results presented in the table are for AUGs that don't have additional AUG 50 nucleotides upstream and downstream.*

Supplementary results

Estimating the effect of non-AUG codon on SSU RC

From table S4 we can see that the MIC score for AUGs with high context score is the highest obtained between the codons that were tested.

For “CUG” codon the MIC results are relatively high, compared to the other AUG-like codons. However, compared to the AUG codon, we cannot see the trend of the SSU lingering upstream to the codon (Figure S10). For the “AGG” codon, we do see an accumulation of RC before the codon, but there is not much of a difference between high and low context score, while for the “AUU” codon we didn’t observed any trends, and the high and low context score cases do not differ in their MIC score (Figure S10).

Examining all codons, not only AUG and AUG-like codons, we do see additional codons with relatively high MIC score , such as CAG. Nevertheless, the difference between CAG with high AUG context score and CAG with low AUG context score is smaller compared to the AUG codon (Figure S11).

For AUG-like codons with short/long distance from the nearest stop codon, the MIC score obtained for long uORFs is usually higher than for short uORFs. However, we didn’t identify RC accumulation surrounding these codons.

The analysis above emphasizes that the visualization of the results in these cases is very informative. The MIC score by itself is indicative to sort the results by their similarity (e.g., as we stated- the RC distribution surrounding AUGs with high context score is more similar to distribution surrounding the main AUG codon, compared to AUGs with low context score), but it must come with the plot of the RC distributions.
